# The effects of ideological value framing and symbolic racism on pro-environmental behavior

**DOI:** 10.1038/s41598-021-00329-z

**Published:** 2021-11-12

**Authors:** Kinga Makovi, Hannah Kasak-Gliboff

**Affiliations:** Social Science Division, New York University Abu Dhabi, Abu Dhabi, UAE

**Keywords:** Psychology and behaviour, Sustainability

## Abstract

**Abstract:**

Environmental degradation continues to be one of the greatest threats to human well-being, posing a disproportionate burden on communities of color. Environmental action, however, fails to reflect this urgency, leaving social-behavioral research at the frontier of environmental conservation, as well as environmental justice. Broad societal consensus for environmental action is particularly sparse among conservatives. The lack of even small personal sacrifices in favor of the environment could be attributed to the relatively low salience of environmental threats to white Americans and the partisan nature of environmentalism in America. We evaluate if (1) environmental action is causally related to the ideological value framing of an environmental issue; and (2) if the perceived race of impacted communities influences environmental action as a function of racial resentment. With this large-scale, original survey experiment examining the case of air-pollution, we find weak support for the first, but we do not find evidence for the second. We advance our understanding of environmental justice advocacy and environmental inaction in the United States.

**Protocol registration:**

The stage 1 protocol for this Registered Report was accepted in principle on 10 June 2021. The protocol, as accepted by the journal, can be found at 10.6084/m9.figshare.14769558.

## Introduction

The simultaneous urgency and scarcity of environmental action, and the social inequalities that result, highlights the question of how to elicit environmental action. While environmental concern has been linked to the intent to act environmentally and a reported willingness to make economic sacrifices for the environment^1^, it does not fully explain patterns of environmental behavior^2^. There is a necessary change in focus away from mere concern towards the environment, and how ideology might shape this relationship^3^. Though numerous models have attempted to explain the determinants of environmental behavior, the attitude-action gap remains: concern in itself is not sufficient to reliably predict environmental action^4^. As such, social-behavioral research has presented itself as a promising avenue for discovering constructive approaches to achieving environmental action, as well as environmental justice by reducing inequalities in the impact of environmental degradation.

A promising area of exploration for a wider uptake of environmental action, which has widely been labeled as a liberal issue, is the use of value framing or “moral reframing” to broaden the appeal of environmental action to conservative audiences^5^. Currently, environmental messages not only do not appeal to conservatives, but have been shown to actively repel them from environmental decisions, such as supporting energy efficiency^6^. Political ideology in itself has been conceptualized as a means of deriving social ontologies, where more ideologically inclined people have consistent views even on issues that do not directly relate to the substance of their ideology^7^. Among conservatives, a skeptical or adversarial stance towards environmentalism could be an example of ontologies at play. Reframing environmental messaging in terms of conservative values has been shown to shrink the difference in environmental attitudes between liberals and conservatives, and therefore shows promise as a method to also increase environmental action^8^.

This paper is situated in the persistent and disturbing trend that the social impacts of environmental degradation disproportionately burden marginalized communities, particularly communities of color, globally and in the United States. The phenomenon has been labeled as environmental injustice or environmental racism, which manifests itself as increased exposure and increased vulnerability to air, water, and soil pollution, which environmentally privileged communities are largely exempt from^9, 10^. Environmental injustice and environmental privilege are two sides of the same coin: access to a clean and safe environment is available only to some, usually white, Americans^10, 11^. Understanding environmental injustice as such raises the question as to whether environmental concern or environmental action can therefore be predicted by racial resentment. Building on Park and Pellow’s framework of environmental privilege, the willingness to take environmental action, or to make a small personal sacrifice for the benefit of the environment and the people effected by it, can be understood as a willingness to pay for a different community’s access to a healthy and safe environment. Therefore, and in accordance with a small body of literature, we anticipate that racial resentment will stifle environmental action^12, 13^, as relatively environmentally privileged white Americans would be less inclined to support a predominantly Black community’s environmental well being^10^.

As of yet, the majority of research related to environmental justice has focused on health and economic challenges as outcomes of racist and unequal systems^14^, however a small set of studies have also examined racism as a potential suppressor of environmental concern^12, 15, 16^. Benegal found that racial resentment correlated with reduced concern over climate change, particularly after Obama was elected president, indicating a “spillover of racialization” suggesting racial resentment as a cause of environmental indifference. Chanin found that racial resentment or symbolic racism predicted lower indices of self reported environmental concern based on data from the General Social Survey^16^, and Dietz and colleagues found that racial resentment also predicted lower donation rates to a water quality organization following the severe water contamination in Flint, Michigan^12^. This paper seeks to further explore the link between racial resentment and environmental indifference, while also utilizing value framing to appeal to audiences across the political spectrum^5, 8, 17^.

To test how value framing, and symbolic racism activated by the perceived race of communities impacted by environmental threats influence environmental action, we designed and fielded an experiment to liberals and conservatives where we manipulate independently the value of environmental protection by invoking conservative or liberal ideology, and the racial composition of the communities being impacted by environmental issues using a personalized story. We focus on a specific form of environmental action: donation to an environmental organization, which can be made at the expense of giving up raffle tickets that respondents could use toward a gift card.

For ease of presentation, we refer to “value aligned” treatments when the intended ideological frame of the treatment aligns with respondents’ ideology, and “value misaligned” treatments, when the opposite is the case. For example, the conservative value framed treatment would be value aligned when assigned to a conservative respondent, and value misaligned when assigned to a liberal respondent. The racial composition of communities impacted was signaled by the names of individuals in a story who have suffered adverse health consequences by air pollution, also referred to as race manipulation through the paper.

We hypothesize that:

### H1

Respondents presented with a value-aligned treatment will donate at higher rates than those presented with a value-misaligned treatment.

We predict that donation rates will vary as a function of value alignment even when controlling for the race manipulation, a measure of environmental concern and symbolic racism in addition to respondent-level controls for gender, age, race, education, income, and ideology. For more detail on these measures, see the “Methods” section.

We also hypothesize that:

### H2

Respondents with high levels of symbolic racism primed with a story about a Black family will donate less.

We predict that donation rates will vary as a function of the interaction between the race manipulation and the symbolic racism score of respondents when controlling for value-alignment, the race manipulation, environmental concern, symbolic racism in addition to respondent-level controls for gender, age, race, education, income, and ideology.

### Experimental design and procedures

Prior to designing and conducting the survey experiment detailed below, we conducted a pilot study that guided the design of our experimental treatments and influenced our proposed analyses. For a detailed description of this pre-test see the relevant section of the Supplementary Information (SI), the main conclusions we derived from it are summarized in the “Methods” section.

Our study proceeds in two steps, i.e., in two separate data collections (for a flow chart, see Fig. S2). In the first data collection, each respondent receives a set of questions that aim to measure symbolic racism and environmental concern, which we treat as baseline measures. At this instance respondents also answer questions about their demographic background and ideology. About three weeks later, in a separate survey, respondents are given the chance participate in a follow-up questionnaire where they are presented with only one of four possible treatments which we randomly assign with equal probability, and are then given the opportunity to donate tokens towards an environmental organization, Earthjustice, or keep the tokens as raffle tickets for a gift card (referred to as: second data collection). This experimental design places respondents in a real-stakes decision-making scenario where they ought to give up a small personal benefit (maximizing their chances of winning the raffle) in order to give resources to an environmental organization.

Each treatment consists of a factual paragraph about air pollution, a short narrative about a family impacted by it, and a paragraph framed to appeal to liberal or conservative values (the full instruments for both data collections are included in the SI). The value frames were designed based on previous research that aimed to prime political ideology^17^. The stories about families only differ in the names of the family members, which were chosen from lists of names typically perceived as Black or as white while keeping social class constant^18^. The primary outcome of the study is respondents’ decision of how many tokens to donate of the 10 they are each awarded, which we operationalize as a continuous outcome (but we also discuss an alternative operationalization as a robustness check). Other key quantities include respondents’ environmental concern which is operationalized as a scale based on^16^, and a measure of symbolic racism, also operationalized as a scale based on^12, 16, 19^. We measure respondents’ political ideology on the basis of respondents’ self reports and the classification used by TurkPrime that provides services to researchers fielding their survey to Amazon Mechanical Turk Workers, but we also discuss alternative operationalizations as robustness checks.

Respondents were recruited via Amazon Mechanical Turk, using the services of TurkPrime (additional details for “Inclusion criteria and data quality” are discussed in the “Methods” section). In total 1582 respondents took part in the first data collection and received $0.50 as a show up fee where we collect information on the variables that form the basis of the symbolic racism measure, and the measure of environmental concern. In the second data collection, about three weeks after the first, 1153 respondents (72.9% of the initial sample) returned, and received $1.00 for completing the survey paid as a show-up fee. In addition, those who answered the comprehension check questions about the text on air-pollution correctly on their first attempt received a bonus of $0.50, which 94.6% of respondents earned. In the second data collection two randomly selected respondents also received a $100 Amazon gift card, to incentivize respondents from the first data collection to participate. Chances to be selected were proportional to the number of tokens that respondents kept, and did not donate to the environmental organization. Respondents know that the tokens that they do not keep will be converted into real-world money and donated to an environmental organization: for each token given to the organization, we donated $0.50 on the respondent’s behalf. On average the first survey took respondents 4.62 mins, the second 7.25 mins. Descriptive statistics for the sample can be found in Table S4, and the distribution of the main outcome measure is presented in Fig. S2.

In order to verify that respondents are categorized by TurkPrime as conservative and liberal accurately, respondents are also asked to identify themselves on a scale ranging from “very liberal” to “very conservative.” We restrict the sample to respondents whose self-identification and TurkPrime classification coincide. Only 8.43% of conservatives and 2.62% of liberals identified differently from the categorization applied by TurkPrime. Note, that we do not have any way of knowing which measure is accurate, and that political ideology of respondents might change over time. The reason why we rely in our main analyses on the categories from TurkPrime as well as self-reports, is because we assume that in these cases we would have the most accurate information on respondents who have a stable, well-formed ideology. We also conduct our main analyses restricting our sample to those who answered comprehension check questions correctly on their first attempt (94.6% of the sample). These individuals, we anticipate, pay closer and more careful attention to the treatment, and are more likely to frame their decisions in these terms. Applying these two exclusion criteria, we analyze responses from 1034 individuals (89.7% of the sample collected in the second data collection). Note that the analytical sample has a virtually identical distribution over demographic characteristics and scores of symbolic racism and environmental concern of all respondents in the first data collection, and all respondents in the second data collection (Table S2), therefore we do not anticipate any of our results to be biased by sample selectivity on the basis of these characteristics.

## Results

In Table 1 we present the results of two regression models, each serving a purpose to test H1 and H2 (Model 1 and Model 2, respectively). We do not find a statistically significant effect of matching ideological value framing on donations. i.e., we fail to uncover evidence consistent with H1. Substantively, in our experiment respondents do not donate more tokens to Earthjustice when the ideological value framing of the text they read aligns with their ideology. We do not find evidence of a statistically significant interaction effect between respondent’s symbolic racism scores and reading a personalized story of a Black family impacting donations either, i.e., we fail to uncover evidence consistent with H2. Substantively, in our experiment respondents with higher symbolic racism scores do not donate significantly lower amounts when they read about a Black (as compared to a white) family impacted by air-pollution. We conduct four set of robustness analyses.

**Table 1:** Modeling donations as a function of value alignment (H1), and as a function of the interaction between symbolic racism and the implied race of the community impacted (H2); additional controls include gender, age, race, education, income and ideology; the complete regression table is presented in Table S3.

	Dependent variable	
	Donation amount	
	Model 1 (H1)	Model 2 (H2)
Value aligned (treatment)	0.293 (0.203)	0.296 (0.203)
Black race (treatment)	0.110 (0.200)	0.212 (0.486)
Environmental concern	4.808*** (0.613)	4.808*** (0.614)
Symbolic racism	− 1.729** (0.816)	− 1.603 (0.982)
Symbolic racism × Black race (treatment)		− 0.235 (1.022)
Constant	− 0.085 (0.843)	− 0.144 (0.881)
Observations	1034	1034
R^2^	0.124	0.125
Adjusted R^2^	0.109	0.108
Residual std. error	3.206 (df = 1015)	3.208 (df = 1014)
F statistic	8.016*** (df = 18; 1015)	7.590*** (df = 19; 1014)

*p < 0.1; **p < 0.05; ***p < 0.01.

First, while our main analysis excludes respondents who failed to answer the comprehension check questions correctly the first time, we also conduct the main analyses on the full sample. With these analyses we aim to show that any findings we might uncover are robust to noise, which would likely increase with the inclusion of less attentive respondents. If anything, the inclusion of these observations we believed would weaken our findings, but might enhance their external validity as in daily life it is unlikely that all casual readers of news and materials produced by awareness campaigns are read with close attention to detail. These robustness analyses (Table 2) reveal consistent findings with those presented in Table 1 for H2, but we do find evidence consistent with H1 (a marginally significant point estimate of 0.331, $p < 0.1$ for value aligned treatments, i.e., an increase in donations of 0.3 of a token). A potential reason for this could be that while inattentive readers may have focused less on facts (which the comprehension check questions asked about), they could have ascertained the ideological message (potentially unconsciously). Note however, that the point-estimates between the models estimated on the restrictive and the full sample are not statistically significantly different.

**Table 2:** Modeling donations as a function of value alignment (H1), and as a function of the interaction between symbolic racism and the implied race of the community impacted (H2); additional controls include gender, age, race, education, income and ideology.

	Dependent variable			
	Donation amount			
	Model 1	Model 2	Model 3	Model 4
Value aligned (treatment)	0.293 (0.203)	0.331* (0.198)	0.296 (0.203)	0.333* (0.198)
Black race (treatment)	0.110 (0.200)	0.038 (0.196)	0.212 (0.486)	0.134 (0.477)
Environmental concern	4.808*** (0.613)	4.846*** (0.595)	4.808*** (0.614)	4.845*** (0.595)
Symbolic racism	− 1.729** (0.816)	− 1.551* (0.794)	− 1.603 (0.982)	− 1.433 (0.958)
Symbolic racism × Black race (treatment)			− 0.235 (1.022)	− 0.221 (1.001)
Constant	− 0.085 (0.843)	− 0.069 (0.806)	− 0.144 (0.881)	− 0.125 (0.846)
Observations	1034	1089	1034	1089
R^2^	0.124	0.120	0.125	0.120
Adjusted R^2^	0.109	0.105	0.108	0.104
Residual std. error	3.206 (df = 1015)	3.210 (df = 1070)	3.208 (df = 1014)	3.211 (df = 1069)
F statistic	8.016*** (df = 18; 1015)	8.109*** (df = 18; 1070)	7.590*** (df = 19; 1014)	7.678*** (df = 19; 1069)

Model 1 and 3 are also presented in Table 1, while Model 2 and 4 repeat the same analysis while including respondents who did not respond to all comprehension check questions correctly on the first attempt.

*p < 0.1; **p < 0.05; ***p < 0.01.

**Table 3:** Modeling donations as a function of value alignment (H1), and as a function of the interaction between symbolic racism and the implied race of the community impacted (H2); additional controls include gender, age, race, education, income and ideology.

	Dependent variable			
	Donation amount			
	Model 1	Model 2	Model 3	Model 4
Value aligned (treatment)	0.293 (0.203)	0.274 (0.197)	0.296 (0.203)	0.275 (0.197)
Black race (treatment)	0.110 (0.200)	0.192 (0.194)	0.212 (0.486)	0.248 (0.476)
Environmental concern	4.808*** (0.613)	4.419*** (0.584)	4.808*** (0.614)	4.419*** (0.584)
Symbolic racism	− 1.729** (0.816)	− 1.909** (0.783)	− 1.603 (0.982)	− 1.842* (0.941)
Symbolic racism × Black race (treatment)			− 0.235 (1.022)	− 0.129 (0.995)
Constant	− 0.085 (0.843)	0.069 (0.801)	− 0.144 (0.881)	0.037 (0.838)
Observations	1034	1091	1034	1091
R^2^	0.124	0.122	0.125	0.122
Adjusted R^2^	0.109	0.107	0.108	0.107
Residual std. error	3.206 (df = 1015)	3.195 (df = 1072)	3.208 (df = 1014)	3.197 (df = 1071)
F statistic	8.016*** (df = 18; 1015)	8.284*** (df = 18; 1072)	7.590*** (df = 19; 1014)	7.842*** (df = 19; 1071)

Model 1 and 3 are also presented in Table 1, while Model 2 and 4 repeat the same analysis while including respondents whose self-reported ideology differed from the classification given by TurkPrime.

*p < 0.1; **p < 0.05; ***p < 0.01.

Second, those whose self-reported ideology did not coincide with the TurkPrime classification are included in this robustness check, and the TurkPrime classification is taken into account. With these analyses we aim to show that any findings we uncover are robust to noise, which would likely increase with the inclusion of all respondents with potentially less solidified ideology and/or less attention to the survey. These analyses are presented in Table 3 and reveal substantively the same result as those obtained in Table 1.

As our pilot data uncovered, the intended race signal of our manipulation has not been uniformly understood by respondents, and interacted with the ideology treatment. We therefore conduct two sets of robustness analyses. It is not possible for us to conclusively decide if respondents are influenced by the amount they donated when answering the follow-up questions about the treatment which includes a question about the perceived race of the family in the story, or if respondents’ assessment of the race of the family is casually influenced by the ideology they attribute to the treatment. Therefore, we conduct the main analyses by restricting the sample in two ways. Both of these robustness checks might yield more accurate results (particularly for H2), however, these may not rise to the level of statistical significance due to smaller samples.

First, we restrict the analytical sample to respondents who identified the race manipulation as we intended. We anticipated that these analyses might yield stronger results, but it also reduces our sample size. Indeed, our sample size reduced to only 617 respondents (59.7% of the analytical sample), and the results are presented in Table 4. While we do not observe a statistically significant interaction effect, the size of the effect is considerably larger in absolute value (a point estimate of − 0.234 compared to a point estimate of − 1.324). Second, we repeat the main analyses by treating respondents’ understanding of the race manipulation, i.e., their self-reports of the race of the family, and taking that as the signal. Note that in this particular case only respondents who identified families as non-Hispanic white and families as Black enter our analyses (i.e., 778 individuals, 75.2% of the analytical sample). These models are presented in Table 5, and reveal substantively similar findings to those presented in Table 1. In this case as well while we do not observe a statistically significant interaction effect, the size of the effect is considerably larger in absolute value (a point estimate of − 0.234 compared to a point estimate of − 1.523). We return to this point in the discussion.

**Table 4:** Modeling donations as a function of value alignment (H1), and as a function of the interaction between symbolic racism and the implied race of the community impacted (H2); additional controls include gender, age, race, education, income and ideology.

	Dependent variable			
	Donation amount			
	Model 1	Model 2	Model 3	Model 4
Value aligned (treatment)	0.293 (0.203)	0.159 (0.263)	0.296 (0.203)	0.163 (0.263)
Black race (treatment)	0.110 (0.200)	0.192 (0.257)	0.212 (0.486)	0.764 (0.620)
Environmental concern	4.808*** (0.613)	4.661*** (0.790)	4.808*** (0.614)	4.655*** (0.790)
Symbolic racism	− 1.729** (0.816)	− 1.894* (1.025)	− 1.603 (0.982)	− 1.157 (1.257)
Symbolic racism × Black race (treatment)			− 0.235 (1.022)	− 1.324 (1.305)
Constant	− 0.085 (0.843)	0.598 (1.079)	− 0.144 (0.881)	0.239 (1.136)
Observations	1034	617	1034	617
R^2^	0.124	0.138	0.125	0.140
Adjusted R^2^	0.109	0.112	0.108	0.113
Residual std. error	3.206 (df = 1015)	3.173 (df = 598)	3.208 (df = 1014)	3.173 (df = 597)
F statistic	8.016*** (df = 18; 1015)	5.337*** (df = 18; 598)	7.590*** (df = 19; 1014)	5.111*** (df = 19; 597)

Model 1 and 3 are also presented in Table 1, while Model 2 and Model 4 represent a sample restricted to those who correctly identified the race treatment as signaled (i.e., as Black or as white).

*p < 0.1; **p < 0.05; ***p < 0.01.

**Table 5:** Modeling donations as a function of value alignment (H1), and as a function of the interaction between symbolic racism and the implied race of the community impacted (H2); additional controls include gender, age, race, education, income and ideology.

	Dependent variable			
	Donation amount			
	Model 1	Model 2	Model 3	Model 4
Value aligned (treatment)	0.293 (0.203)	0.146 (0.233)	0.296 (0.203)	0.141 (0.233)
Black race (treatment)	0.110 (0.200)		0.212 (0.486)	
Black race (perceived)		0.092 (0.231)		0.754 (0.551)
Environmental concern	4.808*** (0.613)	4.294*** (0.705)	4.808*** (0.614)	4.285*** (0.704)
Symbolic racism	− 1.729** (0.816)	− 1.598* (0.924)	− 1.603 (0.982)	− 0.793 (1.105)
Symbolic racism × Black race (treatment)			− 0.235 (1.022)	
Symbolic racism × Black race (perception)				− 1.523 (1.150)
Constant	− 0.085 (0.843)	0.770 (0.970)	− 0.144 (0.881)	0.402 (1.008)
Observations	1034	778	1034	778
R^2^	0.124	0.116	0.125	0.118
Adjusted R^2^	0.109	0.095	0.108	0.096
Residual std. error	3.206 (df = 1015)	3.185 (df = 759)	3.208 (df = 1014)	3.183 (df = 758)
F statistic	8.016*** (df = 18; 1015)	5.513*** (df = 18; 759)	7.590*** (df = 19; 1014)	5.321*** (df = 19; 758)

Model 1 and 3 are also presented in Table 1, while Model 2 and Model 4 represent a sample restricted who identified the family either as Black or white, using these perceptions as the race treatment.

*p < 0.1; **p < 0.05; ***p < 0.01.

Our last robustness analysis concerns the functional form for modeling donations. We repeat our main analysis using a binary outcome variable (donated or not), and a Tobit specification—both have been used in prior work^20, 21^ demonstrating that dictator games typically produce skewed distributions, which our design mimics. These alternatives might be more appropriate functional forms to consider, which is important, as incorrect functional forms may induce biased estimates. We anticipated that the main conclusions from these analyses will broadly align with the main specifications, which is indeed the case as presented in Table 6.

**Table 6:** Modeling whether or not respondents donated any amount as a function of value alignment (H1), and as a function of the interaction between symbolic racism and the implied race of the community impacted (H2); additional controls include gender, age, race, education, income and ideology.

	*Dependent variable:*			
	If Donated			
	Model 1 (H1 logit)	Model 2 (H2 logit)	Model 3 (H1 Tobit)	Model 4 (H2 Tobit)
Value aligned	0.085 (0.144)	0.081 (0.144)	0.085 (0.144)	0.081 (0.144)
Environmental concern	2.611*** (0.445)	2.610*** (0.445)	2.611*** (0.445)	2.610*** (0.445)
Symbolic racism	− 0.476 (0.569)	− 0.741 (0.690)	− 0.476 (0.569)	− 0.741 (0.690)
Black race treatment	0.066 (0.142)	− 0.160 (0.360)	0.066 (0.142)	− 0.160 (0.360)
Symbolic racism × Black race (treatment)		0.496 (0.727)		0.496 (0.727)
Constant	− 1.669*** (0.587)	− 1.538** (0.618)	− 1.669** (0.587)	− 1.538** (0.618)
Observations	1034	1034	1034	1034
Log Likelihood	− 591.334	− 591.101	− 591.334	− 591.101
Akaike Inf. Crit.	1220.669	1222.203	1220.669	1222.203

Model 1 and 2 employs a logistic regression, while Model 3 and 4 a Tobit specification.

*p < 0.1; **p < 0.05; ***p < 0.01.

### Exploratory analysis

In exploratory analyses we aim to document any heterogeneity in the effects in H1 and H2 by political ideology and environmental concern. These analyses are particularly important for developing future work. While the liberal and conservative manipulations are interpreted significantly differently on a 7-point conservative-liberal scale (differences in means 0.380 in the pooled sample, $p < 0.001$, 0.308 among conservatives, $p < 0.1$, and 0.455 among liberals, $p < 0.001$) our ideology treatments are both considered in mostly liberal terms (consistent with the conclusions derived in the pre-test). Therefore, we anticipated that the conservative treatments might be less effective, and in particular, conservatives may not read the intended value-aligned treatment in conservative terms. We therefore explore treatment effect heterogeneity by political ideology by estimating the same regressions for liberal and conservative respondents separately (as defined for the main analyses). Indeed, we find that value alignment is significantly associated with donations amongst liberals (point estimate of 0.561, $p < 0.1$), but not amongst conservatives (point estimate of 0.046, $p = 0.867$), see Table S4. This could be a shortcoming of our treatment (e.g., a longer text may have emphasized ideology more), could also be a result of the politicized nature of environmental action in the US^8^, or may have revealed true heterogeneity of effects. Future work should explore these avenues to enhance the efficacy of environmental communication.

Additionally, we also explore if those with high levels of environmental concern donate different amounts when they read a value aligned treatment. A positive and statistically significant coefficient would mean that value alignment may elicit environmental action amongst those who are already concerned about the environment. Alternatively, a negative and statistically significant coefficient would mean that value alignment may elicit environmental action amongst those who have not been concerned about the environment, and therefore provides us with a powerful tool for intervention to nudge those towards environmental action who are not predisposed to be engaged in it. We, however, do not find a significant interaction between environmental concern and value alignment (Table S5).

Last but not least we also conduct additional analysis in Table S6 with a series of variables focusing on (1) respondents experiences about the text (specifically, if the text made them feel that change was possible, weather or not they found the text as (ideologically) biased, if they found the facts presented to be credible, and the tone of the text ranging from negative to positive); (2) respondents’ views of the family presented (using two operationalizations: if they found the family to be relatable, or if they would invite the family for dinner); (3) if others who hold similar ideological views (in case of liberals, other liberals, in case of conservatives, other conservatives) respondents believe are concerned about the environment and race-based inequality; (4) the environmental impact respondents perceive their families and communities face. The two operationalizations for respondents’ perceptions of the family presented in the story are used in separate regression. We uncover the following associations (evaluated on the pooled sample): respondents who perceive the text to be hopeful about the possibility of change contribute more (point estimate 0.227, $p < 0.05$), while those who perceive it to be biased contribute less (point estimate − 0.190, $p < 0.05$). We find that respondents who would have invited the family for dinner also contribute more (point estimate 0.125, $p < 0.1$). Respondents concern that their communities are impacted by environmental degradation also contribute more (point estimate 0.272, $p < 0.05$). Some of these associations appear to be driven by only one group of respondents by political ideology. Note that these additional variables do not weaken the impact of environmental concern of respondents on donations, and also that the effect sizes are significantly smaller than that of environmental concern. These explorations ought to be interpreted with caution. This analysis was not designed to establish causal relationships; nor should null-effects be interpreted as evidence of no association, and no potential causal effect. For example, the measures of respondents’ beliefs over how other ideologically similar people think about the environment is consistently and positively associated with donations in models that use only these additional factors. This variation is likely a result of collinearity issues, and should not be taken as evidence that norms do not matter for environmental action.

## Discussion

Based on a large, original survey experiment conducted amongst US liberals and conservatives we failed to find evidence consistent with our two hypotheses. Specifically, ideological alignment with respondents’ political ideology of a text on air pollution did not enhance environmental action, nor did symbolic racism moderate the effect of reading a personal story about a Black family on environmental action. While the treatments we implemented did not achieve the anticipated effects, these results, coupled with our observations gleaned from robustness and exploratory analyses provide us with invaluable lessons for environmental communication.

Why did we not uncover an impact of ideological value framing on environmental action? One of the most likely explanations may lie in the weakness of the proposed treatments. As we uncovered during the pre-test and confirmed in the main study, the ideological leaning of all our treatments have been perceived mostly in liberal terms. As a consequence, the actual sample size: respondents who were treated as intended, is much smaller compared to the size of our analytical sample. In case of smaller effects, this reduction of sample size may have resulted in our study being under-powered. As we outlined in our conclusions for the pre-test, however, strengthening the ideological value-frame would have required lengthening the text with ideological messaging, which would have compromised the external validity of our approach. Future work might explore the combination of other types of media to convey ideology via photos or a short video, which could accompany real-world environmental messages. These visual approaches could be more effective in enhancing environmental action, however, they are challenging to design in a way that would not introduce unintentional effects. Alternatively, a liberal or conservative leaning news paper or broadcaster could be associated with the text. This would make the intended ideological leaning of the message more explicit, however such an approach would involve deception. The use of actual messages may compromise the level of similarity in coverage of the same, real-world events. Deception could result in raising respondents’ suspicion about the veracity of what they read (and/or might question if the source indeed produced the text) and influence their environmental action as a result (note that respondents who believed the text they read was ideologically biased donated less). Last but not least, it is important to note that we do find statistically significant effects of ideological value framing amongst liberals. The reasons for this could both be the already-mentioned ones; or they could result from heterogeneous effects as mentioned earlier. Which of these might be the explanation should be subject of future work.

Why did we not uncover an interaction effect between respondents’ symbolic racism scores and them reading a story about a Black family suffering from the consequences of air pollution? Our robustness analyses reveal some potential clues. Specifically, we found this effect increases meaningfully in size when we restricted our analyses to respondents who correctly identified the intended race of the family. Why would respondents misidentify the race of the family and why would this interact with the intended ideological frame is unclear. Future work should however, be particularly attentive to this observation as such effects might interfere with the efficacy of ideological value frames. One potential explanation may be that liberal ideology that prioritizes principles of equity and re-distributive justice may automatically be assumed^22^. If true, this could create feelings of resentment and hostility as political ideology is linked to an increasingly divisive identity arranged along party-lines in the US and beyond^23, 24^. It is also possible that the narrative of communities of color being disproportionately impacted by environmental risks already primed respondents outside of the context of the survey experiment, and that their donations were based more in their pre-existing notions of environmental risk. Note however, that our measure of symbolic racism is consistently, and negatively associated with environmental action in almost all the models we have estimated. It is therefore possible that priming respondents with a story of the environment, regardless of the race manipulation, may have already elicited lower levels of environmental action as a result of resentment about race.

In closing, we highlight that our design may have unintentionally elicited environmental action mostly as a result of conscious decisions rooted in rational choice. To encourage thoughtful decisions and careful attention to detail, respondents were asked comprehension check questions prior to making their decisions. This is standard practice in experimental behavioral research, and in the paradigm of behavioral economics only decisions should be considered and analyzed where researchers have been able to verify that subjects understood the payoff structure of economic games. However, people may make most of their daily decisions as a result of their habits, including whether or not to recycle, or whether or not to donate a small amount for environmental causes when opportunities arise as part of routine activities. To an extent, our design decision to ask comprehension check questions and restrict the analytical sample to those who have fully understood the text on a first try may have resulted in more calculated (and potentially less externally valid) decisions^25^. This observation is particularly important for future experimental work and requires further investigation.

Moving the needle on environmental action, which is equally effective on both sides of the ideological divide is essential. The consistent positive association of concern and action might be one way to strengthen peoples’ will to act on behalf of the environment. However, prior work has already shown the limits of such approaches. Our exploratory analyses revealed that there may be several other pathways, such as an emphasis one’s community environmental risk and ensuring unbiased communication about environmental harms. Future work might focus on these correlates to see how they might be manipulated, and their causal effects established on environmental action. Our study highlighted that possibly different levers need to be applied on the two sides of the ideological divide.

We attempted to quantify the effect of value framing on environmental action, by using key words and concepts central in conservative and liberal discourse. What we found however, suggests that effective messaging must do more than adopting already existing strategies. The association of environmentalism with liberal ideologies appears to overwhelm our attempts to create an environmental message that is understood in conservative terms. The challenge ahead may lie not only in our framing of environmental justice, but also in invoking the identities and norms of the target audiences. For respondents to be spurred to action by any messaging, they might need to image others with whom they identify and share values to take action. Coherence between the message and intended ideology is paramount, not only for minimizing perceived bias, but also for reinforcing a social norm of environmentalist thought and action among conservatives and liberals alike, in spite of their ideological divide in contemporary America.

## Methods

### Pre-test

In order to ensure that our treatments provide only the intended priming effects, we collected pre-test data. The pre-test helped us to evaluate (1) how the treatment texts are perceived by liberals and conservatives; (2) weather the race and value framing manipulations interact; and (3) how the environmental organization we selected for the main experiment is perceived, respectively.

From our pre-test data collection which we conducted on the same platform, using the same data quality filters, we drew the following conclusions. First, the choice of the organization appears to work for our purposes: it is an organization whose work was regarded as relevant for environmental action, and most respondents believed it was effective or highly effective; while an overwhelming majority of respondents have not heard of Earthjustice. In sum, we found no evidence in our pre-test that the selection of the organization interacted with the treatments in unanticipated ways, or that respondents had significant doubts about the relevance or effectiveness of the organization which could influence donations in unanticipated ways.

The pre-test also revealed that the treatments’ use of ideological and race signals are not orthogonal, given the politicized nature of race in America. This interaction appears to us to be virtually unavoidable, and motivated us to include measures in the study on how the race manipulation is perceived by respondents. We opted to include these measures in the main study that allow us perform exploratory analyses using the answers to these questions. While it is possible that motivated reasoning after making donations could influence these answers, we perform analyses on a restricted sub-sample namely on those respondents who correctly identified the intended race of the family. These analyses were suggested as a result of our analyses of pre-test data.

As the pre-test uncovered that the ideology prime was weak, we strengthened the treatment by adding an additional sentence to the paragraph priming of ideology compared to what we used in the pre-test. While based on prior work a superior approach would have been to use a longer value-frame, we opted for the current approach to accommodate the race-prime and to give both primes equal weight in the treatment we designed given the research questions for this study. Additionally, a longer value-frame would have compromised the external validity of the treatments that are designed to present respondents with how a typical news story about air pollution or awareness campaign message might read.

### Inclusion criteria and data quality

Respondents must be age 18 or over, based in the United States with at least 100 Human Intelligence Tasks (HIT) completed with at least a 95% approval rating as well as agree to the terms outlined in the consent form in order to participate. Respondents were selected from among CloudResearch Approved respondents (https://www.cloudresearch.com/resources/blog/new-tools-improve-research-data-quality-MTurk/) to further enhance data quality, whose ideology was assessed by TurkPrime, and who identified as “very conservative,” “conservative,” “liberal,” or “very liberal.”

In addition to the already outlined restrictions that influence who may see the HIT, we also used multiple screens to avoid the same individual participating in the study more than one time, making sure that all respondents in the study see only one of the experimental conditions. First, we created a survey group within TurkPrime for all HITs associated with this study, including the pre-test. The same MTurk worker cannot take multiple surveys in the same TurkPrime survey group. Second, we activated the “ballot box stuffing” option in Qualtrics, to prevent multiple entries from the same IP address. In addition, we sequentially compiled a list of the MTurk IDs of workers who enter the study using an external SQL database and we automatically verify that each new MTurk worker had not already been part of this list. Our sample, therefore, is limited to unique individuals who took part in the study once, which includes the pre-test data, i.e., respondents in the pre-test study were not eligible to participate in the main study so that we assure all respondents have seen the text about air-pollution the first time.

The fielding for the first data collection took place between January 24th and February 4th, 2021, while the second data collection was carried out between March 9h and March 23rd, 2021.

### Ethics Information

The consent form provided to respondents gave a brief description of the content of the survey and how the respondent will be compensated. Informed consent was obtained online. The study was approved by the NYU Abu Dhabi Internal Review Board (#HRPP-2020-119), and was deemed minimal risk. Our study does not involve deception or the collection of sensitive information, but we did collect personal identifiers (MTurk IDs). The personal identifiers were used to award respondents bonuses and to match respondents’ answers to each of the two parts of the survey.

All methods were performed in accordance with the relevant guidelines and regulations, and were executed according to the pre-registration.

### Control variables

Our regression models include controls for the following variables: gender, age, race, education, and income. We discuss their operationalization in-turn. We operationalize gender as female or non-female (a category which contains other than the male-female gender binary). Age is binned: 18-25, 26-45, 46-65, 66 and up. Race is measured as white or non-white, where white are all respondents who did not identify with any other racial group and/or Hispanic ethnicity. Education was operationalized as a categorical variable of: high school or less, some college (including those with associate degrees), undergraduate degree, or graduate degree. Income is also binned with the following five categories and one for those who withheld their income: less than $24,999; $25,000-$49,999, $50,000-$74,999, $75,000-$150,000, more than $150,000.

## Supplementary Information

Supplementary Information.

Supplementary Dataset.

R Scripts.

## Data Availability

We deposited the data for the pilot on Open Science Framework (https://osf.io/7cxe9/), alongside the data for the main study.

## Code availability

We deposited the code for the pre-test on Open Science Framework (https://osf.io/7cxe9/), alongside the code for the main analyses.
