# Supplementary material for: The effects of ideological value framing and symbolic racism on pro-environmental behavior
Source: Sci Rep. 2021 Nov 12;11:22189. doi: 10.1038/s41598-021-00329-z (PMC8589981; doi:10.1038/s41598-021-00329-z)
Supplement: Supplementary file 1 — Supplementary Information. [file 41598_2021_329_MOESM1_ESM.pdf]

Supplementary Information: The Effects of Ideological Value Framing and Symbolic Racism on Pro-Environmental Behavior

[1,+,*]Kinga Makovi [1,+]Hannah Kasak-Gliboff
[1]New York University Abu Dhabi, Social Sciences, Abu Dhabi, UAE
[+]These authors contributed equally to this work.
[*]Corresponding: km2537@nyu.edu

Flow Chart of the Experiment

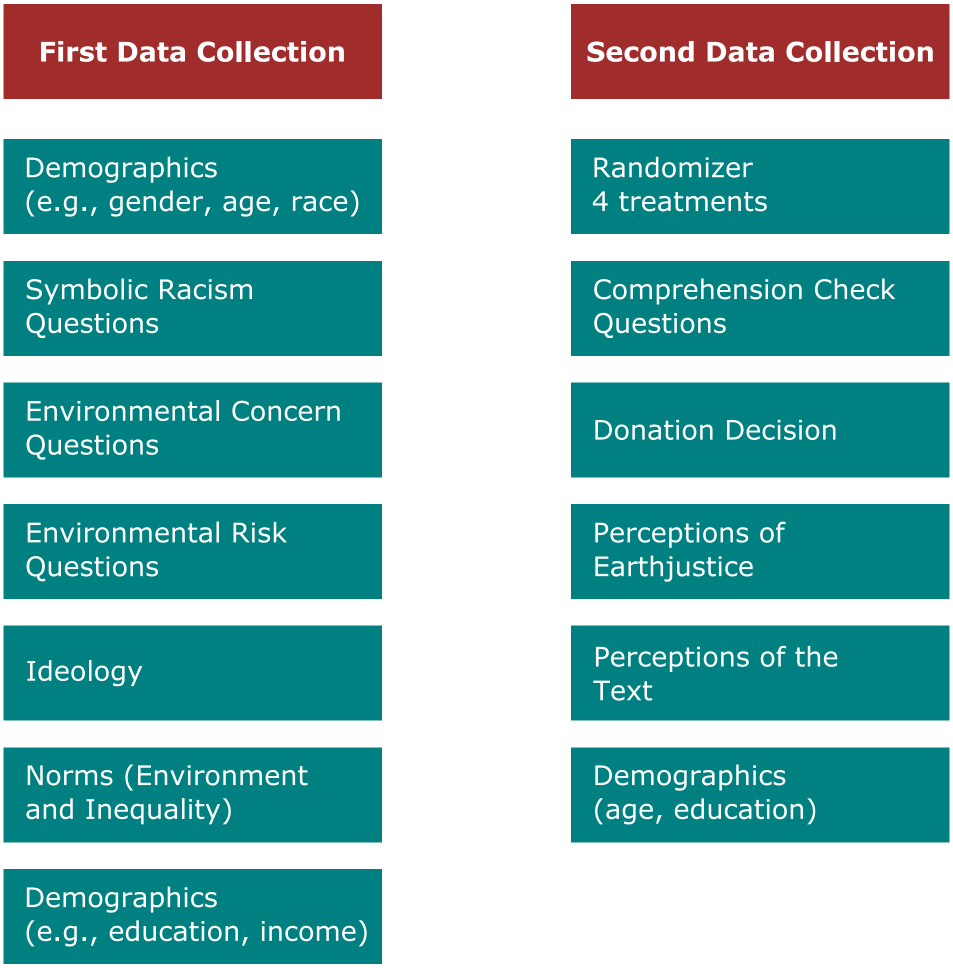

Figure S2. Flow chart of the experiment, describing the order in which respondents saw the questions, and which data collection were they presented.

Descriptive Statistics

Table S2. Descriptive statistics of the first and second data collections and the analytical sample.

	Wave 1<br>N = 1,582	Wave 2<br>N = 1,153	Analytical Sample<br>N = 1,034
**Age Range**			
18-25	140 (9)	84 (7)	66 (6)
26-45	894 (57)	604 (52)	541 (52)
46-65	427 (27)	357 (31)	325 (31)
66 and up	121 (8)	108 (9)	102 (10)
**Race**			
White	1,314 (83)	964 (84)	869 (84)
Non-White	268 (17)	189 (16)	165 (16)
**Gender**			
Female	833 (53)	610 (53)	555 (54)
Non-Female	749 (47)	543 (47)	479 (46)
**Income**			
Lowest Quintile	210 (13)	140 (12)	124 (12)
Second	402 (25)	292 (25)	264 (26)
Third	371 (23)	281 (24)	258 (25)
Fourth	468 (30)	344 (30)	308 (30)
Highest Quintile	111 (7)	81 (7)	70 (7)
Prefer Not to Answer	20 (1)	15 (1)	10 (1)
**Education**			
High School or Less	149 (9)	113 (10)	95 (9)
Some College	471 (30)	331 (29)	293 (28)
Undergraduate Degree	675 (43)	482 (42)	441 (43)
Graduate Degree	287 (18)	227 (20)	205 (20)
**Ideology (TurkPrime Classified)**			
Conservative	793 (50)	581 (50)	506 (49)
Liberal	789 (50)	572 (50)	528 (51)
**Ideology (Self-Reported)**			
Conservative	717 (45)	533 (46)	506 (49)
Liberal	795 (50)	572 (50)	528 (51)
**Environmental Concern**			
Mean (sd)	0.58 ± 0.24	0.58 ± 0.24	0.58 ± 0.24
**Symbolic Racism**			
Mean (sd)	0.44 ± 0.19	0.44 ± 0.19	0.43 ± 0.20

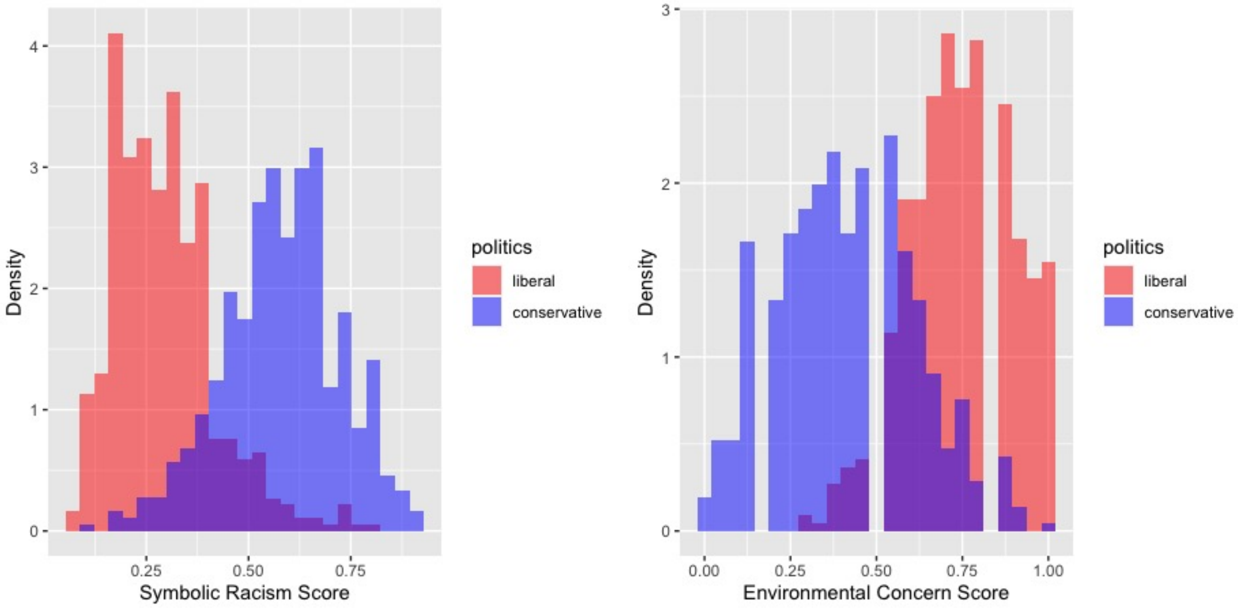

Figure S3. Histograms of Environmental Concern and Symbolic Racism among Liberals and Conservatives

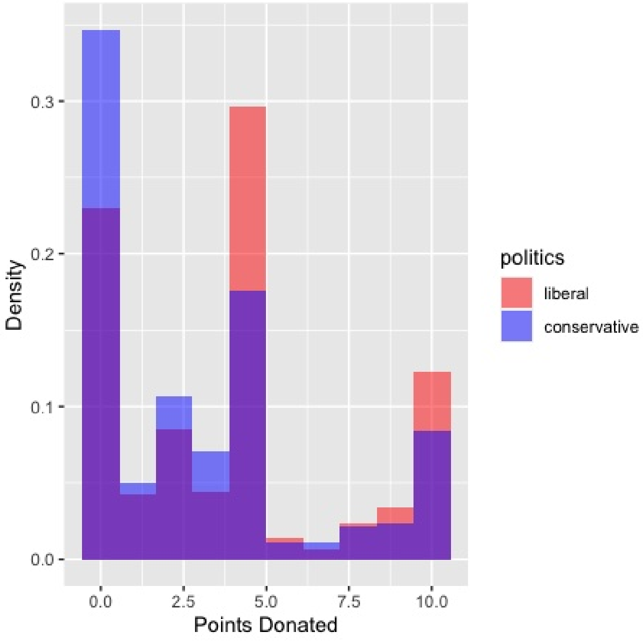

Figure S4. Histogram Showing the Distribution of Donations by Liberals and Conservatives

Full Regression Table

Table S3. Complete regression table for Table 1.

	Dependent variable:	
	Donation Amount	
	Model 1 (H1)	Model 2 (H2)
Value Aligned (Treatment)	0.293	0.296
	(0.203)	(0.203)
Black Race (Treatment)	0.110	0.212
	(0.200)	(0.486)
Environmental Concern	4.808***	4.808***
	(0.613)	(0.614)
Symbolic Racism	-1.729**	-1.603
	(0.816)	(0.982)
Age 26-45	0.193	0.195
	(0.427)	(0.427)
Age 46-65	0.510	0.512
	(0.446)	(0.447)
Age 66 and up	1.139**	1.139**
	(0.524)	(0.524)
Female	0.101	0.101
	(0.204)	(0.204)
Non-White	0.100	0.100
	(0.281)	(0.282)
Some College	0.308	0.308
	(0.386)	(0.386)
Undergraduate Degree	-0.275	-0.274
	(0.377)	(0.377)
Graduate Degree	0.061	0.061
	(0.423)	(0.423)
Second Income Quintile	0.657*	0.659*
	(0.352)	(0.352)
Third Income Quintile	0.419	0.422
	(0.358)	(0.358)
Fourth Income Quintile	0.659*	0.661*
	(0.356)	(0.357)
Highest Income Quintile	0.120	0.124
	(0.500)	(0.500)
(Income) Prefer not to answer	-0.836	-0.831
	(1.064)	(1.065)
Conservative Ideology	0.929***	0.927***
	(0.322)	(0.322)
Symbolic Racism × Black Race (Treatment)		-0.235
		(1.022)
Constant	-0.085	-0.144
	(0.843)	(0.881)
Observations	1,034	1,034
R^2	0.124	0.125
Adjusted R^2	0.109	0.108
Residual Std. Error	3.206 (df = 1015)	3.208 (df = 1014)
F Statistic	8.016*** (df = 18; 1015)	7.590*** (df = 19; 1014)
Note:		*p<0.1; **p<0.05; ***p<0.01

Exploratory Analyses

In Table S4, the analysis of H1 and H2 is broken down into subgroups of liberals and conservatives. The effect of value alignment is statistically significant among liberals, but not among conservatives. Table S5 summarizes the effects of value alignment, environmental concern, symbolic racism, a Black race treatment, and the interaction effect between value alignment and environmental concern. The interaction effect between value alignment and environmental concern is not statistically significant. In Table S6 we include regression result with a large set of additional controls.

Table S4. Modeling donations for liberals (Model 1 and 3) and conservatives (Model 2 and 4) separately as a function of value alignment (H1—Models 1 and 3), and as a function of the interaction between symbolic racism and the implied race of the community impacted (H2—Models 2 and 4); additional controls include gender, age, race, education, and income.

	Dependent variable:			
	Donation Amount			
	Model 1 (Liberal)	Model 2 (Conservative)	Model 3 (Liberal)	Model 4 (Conservative)
Value Aligned (Treatment)	0.561*	0.046	0.558*	0.046
	(0.301)	(0.275)	(0.301)	(0.276)
Black Race (Treatment)	0.017	0.273	0.632	0.286
	(0.296)	(0.272)	(0.767)	(1.143)
Environmental Concern	3.777***	5.318***	3.819***	5.318***
	(1.060)	(0.749)	(1.061)	(0.751)
Symbolic Racism	0.087	-3.138***	1.148	-3.126**
	(1.305)	(1.032)	(1.787)	(1.429)
Symbolic Racism × Black Race (Treatment)			-2.107	-0.023
			(2.423)	(1.913)
Constant	0.019	1.278	-0.355	1.272
	(1.368)	(1.151)	(1.434)	(1.283)
Observations	528	506	528	506
R^2	0.059	0.184	0.061	0.184
Adjusted R^2	0.028	0.156	0.027	0.154
Residual Std. Error	3.364 (df = 510)	3.032 (df = 488)	3.365 (df = 509)	3.035 (df = 487)
F Statistic	1.891** (df = 17; 510)	6.482*** (df = 17; 488)	1.827** (df = 18; 509)	6.110*** (df = 18; 487)
Note:				*p<0.1; **p<0.05; ***p<0.01

Table S5. Modeling donations as a function of value alignment (H1) and the interaction between value alignment and environmental concern; additional controls include gender, age, race, education, income and ideology.

	Dependent variable:
	Donation Amount
Value Aligned (Treatment)	-0.294
	(0.527)
Black Race (Treatment)	0.126
	(0.200)
Environmental Concern	4.294^{***}
	(0.747)
Symbolic Racism	-1.696^{**}
	(0.816)
Environmental Concern × Value Aligned (Treatment)	1.011
	(0.838)
Constant	0.197
	(0.874)
Observations	1,034
R^2	0.126
Adjusted R^2	0.109
Residual Std. Error	3.205 (df = 1014)
F Statistic	7.675^{***} (df = 19; 1014)
Note:	*p<0.1; **p<0.05; ***p<0.01

Table S6. The Association of additional variables and donations

	Dependent variable:					
	Donation Amount					
	Pooled (Dinner)	Pooled (Relatable)	Liberal (Dinner)	Liberal (Relatable)	Conservative (Dinner)	Conservative (Relatable)
Value Aligned (Treatment)	0.264	0.275	0.478	0.509*	-0.011	-0.024
	(0.200)	(0.200)	(0.304)	(0.303)	(0.269)	(0.269)
Black Race (Treatment)	0.116	0.139	0.024	0.053	0.209	0.203
	(0.198)	(0.198)	(0.298)	(0.298)	(0.266)	(0.266)
Environmental Concern	3.680***	3.652***	2.893**	2.886**	3.938***	3.851***
	(0.677)	(0.677)	(1.162)	(1.164)	(0.833)	(0.834)
Symbolic Racism	-1.149	-1.354*	0.355	0.165	-1.913*	-2.159**
	(0.812)	(0.811)	(1.317)	(1.306)	(1.027)	(1.029)
Change is Possible	0.227**	0.264***	0.133	0.162	0.250*	0.284**
	(0.092)	(0.093)	(0.134)	(0.134)	(0.133)	(0.134)
Perceived Bias	-0.190***	-0.202***	-0.045	-0.055	-0.321***	-0.340***
	(0.065)	(0.065)	(0.100)	(0.100)	(0.087)	(0.087)
Perceived Credibility	0.011	0.057	0.072	0.082	-0.081	-0.004
	(0.111)	(0.115)	(0.188)	(0.193)	(0.139)	(0.143)
Perceived Tone	-0.0001	-0.001	-0.063	-0.066	0.032	0.048
	(0.089)	(0.089)	(0.140)	(0.140)	(0.118)	(0.118)
Risk to Family	-0.083	-0.056	-0.224	-0.223	0.027	0.072
	(0.110)	(0.111)	(0.171)	(0.175)	(0.144)	(0.145)
Risk to Community	0.272**	0.296**	0.503**	0.532**	0.126	0.152
	(0.131)	(0.132)	(0.220)	(0.219)	(0.163)	(0.164)
Would Invite to Dinner	0.125*		0.130		0.113	
	(0.072)		(0.122)		(0.090)	
Family is Relatable		-0.098		0.022		-0.194
		(0.096)		(0.157)		(0.121)
Others Care - Environment	-0.175	-0.171	-0.138	-0.145	-0.164	-0.150
	(0.196)	(0.196)	(0.341)	(0.341)	(0.235)	(0.235)
Others Care - Race	-0.287	-0.269	-0.010	0.004	-0.360	-0.332
	(0.184)	(0.184)	(0.327)	(0.327)	(0.220)	(0.220)
Constant	-0.371	0.146	-2.178	-1.838	1.887	2.579*
	(1.224)	(1.197)	(2.121)	(2.099)	(1.566)	(1.521)
Observations	1,034	1,034	528	528	506	506
R^2	0.161	0.160	0.083	0.081	0.244	0.246
Adjusted R^2	0.139	0.137	0.036	0.034	0.203	0.205
Residual Std. Error	3.152 (df = 1006)	3.155 (df = 1006)	3.350 (df = 501)	3.354 (df = 501)	2.946 (df = 479)	2.943 (df = 479)
F Statistic	7.172*** (df = 27; 1006)	7.087*** (df = 27; 1006)	1.753** (df = 26; 501)	1.706** (df = 26; 501)	5.952*** (df = 26; 479)	6.001*** (df = 26; 479)

Note: *p<0.1; **p<0.05; ***p<0.01

Pilot Data

The pretest was fielded on November 17th, 2020 among workers on Amazon Mechanical Turk, using the services of TurkPrime that allows us to use a variety of filters enhancing data quality. The same filters were applied as in the main study. In the pre-test a total of 95 conservatives and 98 liberals participated based on their TurkPrime classification.

In order to ensure that our treatments provide only the intended priming effects, we collected pretest data outlined in this section, and discuss the strengths and shortcomings of the pretest. The pretest helps to evaluate (1) how the treatment texts are perceived by liberals and conservatives; (2) weather the race and value framing manipulations interact; and (3) how the environmental organization we selected for the main experiment is perceived, respectively. We designed the pretest to see if the following expectations about the designed manipulations are met:

1. The value frames, in fact, prime ideology, while not influencing other factors that may also influence environmental action, such as the perceived credibility of facts presented in the text.

2. The names in the stories prime respondents to think of specific communities impacted, while they do not impact other factors that may also influence environmental action, such as the perceived credibility of facts presented in the text.

3. The value framing and race-priming texts do not interact in unexpected ways, e.g., specific combinations of value-frames and names in the text do not influence other factors that may also influence environmental action, such as the perceived credibility of facts presented in the text.

4. The perception of respondents about the efficacy of the environmental organization to fight pollution is not influenced by the treatment participants were exposed to.

To reiterate, our main goal is to compare conservatives to other conservatives and liberals to other liberals receiving different treatments, not to compare conservatives and liberals to one another. We do anticipate that political ideology will be associated with respondents' environmental concern and action, as well as their perceptions of the treatment text, but this is not the subject of our inquiry.

The pretest was fielded on November 17th, 2020, among workers on Amazon Mechanical Turk, using the services of TurkPrime that allows us to use a variety of filters enhancing data quality. The survey instrument can be found in the Appendix. As summarized in Tables S7–S8 we used as set of follow-up questions to gauge respondents perceptions of it.

Our initial expectations have been that the tone and credibility of the text would not be influenced by the manipulations, while the political ideology, the potential for change that the text may elicit, and how biased respondents perceived it, would be. We compare means without representing standardized mean deviations for each of these for ease of presentation.

Perception of Value Framing

Although the priming effect remains small, the treatments are generally perceived as liberal and conservative consistently with our intentions. Liberals consistently rated liberal value framed treatments as more liberal and conservative value framed treatments as more conservative. Specifically, liberals rated the liberal treatment as 2.58 and the conservative treatment as 3.19 on a scale of 1–7 ranging from liberal (1) to conservative (7). Notably, conservatives' perception of political ideology varied by treatment and appears to be influenced by the race treatment as well, where the liberal-white (LW) and conservative-black (CB) treatments were rated very similarly as 3.46 and 3.35, respectively. The value framing effect was amplified in the liberal-black (LB) treatment and conservative white treatment (CW), which were rated as 2.71 and 3.91, respectively.

Perception of Passage Tone, Credibility, and Bias

In terms of credibility, respondents found the facts to be mostly credible across treatments, with only slight variations. Conservatives found the CW to be most credible, however liberals surprisingly found CB to be the most credible, with LB as the least credible. We did not expect these results, however the overall difference in credibility is small. Perceived bias results were similar to our expectations, where conservatives found the CW to be the least biased, while liberals found it to be the most biased. The interaction between the race manipulation and perceived political ideology or bias is revealing the politicised nature of the discussion of race in America, which has apparent spillover effects on environmental views[15].

The perceived tone of liberal treatments were reported to be more negative than conservative ones, with LB being the most negative among conservatives and LW being the most negative among liberals. At the same time, respondents reported feeling that change is less possible after reading conservative treatments, despite also rating liberal treatments as more negative. Among liberals, reading the CB treatment made them feel change was least possible, whereas among conservatives the CW treatment made change seem the least possible.

Perception of Race Manipulation

In order to evaluate priming part of the treatment that intended to manipulate the race of the impacted family, we designed questions about how reliably the names signal the intended race, and risk-perceptions to oneself and ones' community as a result of reading this more personalized story. We present results pertaining to these questions in Tables S9–S10. For perceived risk to family/friends and to the community, conservatives both rated the risk of their family and friends highest when presented with the CW treatment, as we expected due to the effects of race and social distance or symbolic belonging[26,27]. Liberals, on the other hand, perceived risk to friends and family to be the greatest when presented with LB, and to the community when presented with LW treatments. While this was not expected, the difference in risk perceptions should not be overstated either, given the sample size of the pretest.

We also evaluate how the race of the family was perceived, and present these findings in Tables S11–S12. Among conservatives, the intended Black family was perceived as Black 75% and 70% of the time by liberal and conservative value frames, respectively. However, the intended White family was perceived as White only 61% of the time when the political ideology signaled was conservative, while the same figure was 70% in case of the liberal value frame. Additionally, 20% conservative respondents in the CB treatment attributed no race/ethnicity to the family, which is more than twice the rate of this answer category chosen in any other treatment. Interesting variation was observed in the case of liberals as well, where Black and white families paired with the liberal treatment were perceived to have no race/ethnicity over 20% of the time, and were much more likely to be perceived as white (79%) when paired with the conservative treatment than when paired with the liberal treatment (62%). For these reasons, we included follow-up questions about these perceptions in the main study, so that we may use them as control variables, as well as allow us to restrict the sample, specifically to respondents who perceived the race-prime as intended.

Perceptions of the Environmental Organization

We selected **Earthjustice** for the organization to feature in the main study. The organization was chosen as they do not exhibit explicitly partisan ideology. Participants would need to distribute tokens between themselves and the organization, and the tokens they keep are proportional to their chances of winning a gift card; while the tokens they give to the organization would be converted to real money and donated to Earthjustice.

The pretest provides some insight into respondents' knowledge and perception of Earthjustice, detailed in Table S13, S14, and S15. Conservatives and liberals did not show substantially differential knowledge about the organization, 78% and 83% of each group having never heard of it, respectively. Both groups found the organization's work relevant, though considerably more liberals considered Earthjustice "very relevent" as opposed to "somewhat relevant." The majority of both liberal and conservative respondents believed that the political ideology of the organization to be "very liberal" or "somewhat liberal,". Finally, liberal and conservative respondents differ quite substantially in their agreement with Earthjustice's mission and their belief in Earthjustice's efficacy. Because our analysis is not built on comparisons between liberals and conservatives, this does not hinder our ability to study differences in donations within the same ideology, however we included these as post-treatment measures in the main data collection so they can be controlled for robustness analyses, and used in exploratory analyses.

Pretest Conclusions

When it comes to the design of the treatments, we drew the following conclusions from the pretest:

1. The treatments' use of ideological and race signals are not orthogonal, given the politicized nature of race in America[15]. This interaction appears to us to be virtually unavoidable, and motivated us to include measures in the study on how the race manipulation is perceived by respondents. We opt to include these measures in the main study that allows us perform exploratory analyses using the answers to these questions. While it is possible that motivated reasoning after making donations could influence these answers, we perform analyses on a restricted sub-sample namely on those respondents who correctly identified the intended race of the family.

2. As the pretest uncovered that the ideology prime was weak, we strengthened the treatment by adding an additional sentence to the paragraph priming of ideology compared to what we used in the pre-test. While based on prior work, and comments we received, a superior approach would be to use a longer value-frame, we opted for the current approach to accommodate the race-prime and to give both primes equal weight in the treatment we designed. Additionally, we felt that a longer value-frame might compromise the external validity of the treatments that are designed to present respondents with how a typical news story about air pollution or awareness campaign message might read.

When it comes to the design of using Earthjustice as an organization, we drew the following conclusions from the pretest:

1. The choice of the organization appears to work for our purposes.

Table S7. Perceptions among Conservatives by treatment in the pretest data.

	Treatment			
	LB	LW	CB	CW
N of respondents	28	24	20	23
The passage seemed... (1=not biased at all, 7=very biased)				
Mean	4.11	4.12	4.25	3.78
SD	1.83	1.70	1.80	1.62
The facts seemed... (1=not credible at all, 7=very credible)				
Mean	5.18	5.08	5.40	5.91
SD	1.56	1.64	1.14	1.00
The passage seemed to lean politically... (1= liberal, 7= conservative)				
Mean	2.71	3.46	3.35	3.91
SD	1.56	1.67	1.63	1.31
The tone of the passage was... (1=very negative, 7=very positive)				
Mean	2.75	3.29	3.65	3.43
SD	1.38	1.27	1.57	1.56
The passage makes me feel that... (1=change is impossible, 7=change is very possible)				
Mean	5.04	5.25	5.20	4.74
SD	1.26	1.07	1.01	1.25
This passage made me feel that my family and friends are... (1=not at all at risk, 7=very much at risk)				
Mean	4.75	4.25	4.70	5.00
SD	1.48	1.57	1.53	1.21
This passage made me feel that the wider community is... (1=not at all at risk, 7=very much at risk)				
Mean	5.25	5.17	5.05	5.39
SD	1.38	1.55	1.39	1.08

Table S8. Perceptions among Liberals by treatment in the pretest data.

	Treatment			
	LB	LW	CB	CW
N of respondents	28	24	20	23
The passage seemed...<br>(1=not biased at all, 7=very biased)				
Mean	2.42	2.46	2.79	3.29
SD	1.74	1.68	1.77	1.85
The facts seemed...<br>(1=not credible at all, 7=very credible)				
Mean	5.79	6.31	6.33	6.00
SD	1.77	0.79	0.87	1.18
The passage seemed to lean politically...<br>(1= liberal, 7= conservative)				
Mean	2.58	2.58	3.12	3.25
SD	1.47	1.06	1.12	1.29
The tone of the passage was...<br>(1=very negative, 7=very positive)				
Mean	3.08	2.85	3.38	3.00
SD	0.83	1.43	1.17	0.72
The passage makes me feel that...<br>(1=change is impossible, 7=change is very possible)				
Mean	5.17	5.38	5.08	5.17
SD	1.55	1.58	1.18	1.09
This passage made me feel that my family and friends are...<br>(1=not at all at risk, 7=very much at risk)				
Mean	5.75	5.54	5.46	5.42
SD	0.94	1.27	1.22	1.25
This passage made me feel that the wider community is...<br>(1=not at all at risk, 7=very much at risk)				
Mean	6.04	6.15	5.67	5.75
SD	0.95	0.83	1.09	0.90

Table S9. Perceptions of family among Conservatives by treatment in the pretest data.

	Treatment			
	LB	LW	CB	CW
N of respondents	28	24	20	23
The family in the passage seemed...<br>(1=not relatable at all, 7=very relatable)				
Mean	5.18	5.50	4.70	5.09
SD	1.47	1.10	1.84	1.65
How willing would you be to have the family in the passage over for dinner?<br>(1=not willing at all, 7=very willing)				
Mean	5.43	6.08	5.10	5.65
SD	1.37	0.97	1.89	1.19

Table S10. Perceptions of family among Liberals by treatment in the pretest data.

	Treatment			
	LB	LW	CB	CW
N of respondents	28	24	20	23
The family in the passage seemed... (1=not relatable at all, 7=very relatable)				
Mean	6.08	6.00	5.54	5.75
SD	0.97	1.13	1.18	1.19
How willing would you be to have the family in the passage over for dinner? (1=not willing at all, 7=very willing)				
Mean	6.12	5.88	5.79	5.50
SD	1.19	1.51	1.67	1.38

Table S11. Perceptions of family's race among Conservatives by treatment in the pretest data.

	Treatment			
	LB	LW	CB	CW
N of respondents	28	24	20	23
What race or ethnicity do you associate with the family mentioned in the passage?				
White (%)	14.3	70.8	5.0	60.9
Black (%)	75.0	16.7	70.0	26.1
Hispanic or Latino (%)	3.6	4.2	5.0	0
Other (%)	0	0	0	4.3
None (%)	7.1	8.3	20.0	8.7

Table S12. Perceptions of family's race among Liberals by treatment in the pretest data.

	Treatment			
	LB	LW	CB	CW
N of respondents	28	24	20	23
What race or ethnicity do you associate with the family mentioned in the passage?				
White (%)	8.3	61.5	20.8	79.2
Black (%)	70.8	11.5	79.2	4.2
Hispanic or Latino (%)	0	3.8	0	4.2
Other (%)	0	0	0	4.2
None (%)	20.8	23.1	0	8.3

Table S13. Knowledge and perception of Earthjustice in the pretest data.

	Conservatives	Liberals
N of respondents	95	98
Have heard of Earthjustice (%)		
Yes	10.53	7.14
Maybe	11.58	9.18
No	77.9	83.67
Perceived Relevance (%)		
Very relevant	55.79	82.65
Somewhat relevant	41.05	17.35
Not very relevant	2.11	0
Not relevant at all	1.05	0
Perceived Ideology (%)		
Very liberal	26.32	37.76
Somewhat liberal	28.42	46.94
Somewhat conservative	7.37	2.04
Very conservative	21.05	0
No political ideology	16.84	13.27
Agreement with Work (%)		
Strongly agree	34.73	82.65
Somewhat agree	56.84	16.33
Somewhat disagree	5.26	1.02
Strongly disagree	3.16	0
Perceived Effectiveness (%)		
Very effective	30.53	45.92
Somewhat effective	62.11	54.08
Not very effective	5.26	0
Not effective at all	2.11	0

Table S14. Perceptions of Earthjustice among Conservatives by treatment in the pretest data.

	LB	LW	CB	CW
N of respondents	28	24	20	23
Have heard of Earthjustice (%)				
Yes	10.71	4.17	10.0	17.4
Maybe	10.71	12.5	15.0	8.7
No	78.6	83.3	75.0	73.9
Perceived Relevance (%)				
Very relevant	53.6	66.7	40.0	60.9
Somewhat relevant	39.3	33.3	60.0	34.8
Not very relevant	3.57	0	0	4.35
Not relevant at all	3.57	0	0	0
Perceived Ideology (%)				
Very liberal	42.9	12.5	30.0	17.4
Somewhat liberal	21.4	37.5	25.0	30.4
Somewhat conservative	7.14	4.17	10.0	8.7
Very conservative	14.3	20.8	25.0	26.1
No political ideology	14.3	25.0	10.0	17.4
Agreement with Work (%)				
Strongly agree	28.6	37.5	25.0	47.8
Somewhat agree	60.7	58.3	60.0	47.8
Somewhat disagree	3.57	4.17	10.0	4.35
Strongly disagree	7.14	4.17	5.0	0
Perceived Effectiveness (%)				
Very effective	17.9	41.7	25.0	39.1
Somewhat effective	75.0	45.8	70.0	56.5
Not very effective	0	12.5	5.0	4.35
Not effective at all	7.14	0	0	0

Table S15. Perceptions of Earthjustice among Liberals by treatment in the pretest data.

	LB	LW	CB	CW
N of respondents	24	26	24	24
Have heard of Earthjustice (%)				
Yes	20.8	3.85	0	4.17
Maybe	16.7	3.85	12.5	4.17
No	62.5	92.3	87.5	91.7
Perceived Relevance (%)				
Very relevant	79.2	76.92	91.7	83.3
Somewhat relevant	20.8	23.1	8.33	16.7
Not very relevant	0	0	0	0
Not relevant at all	0	0	0	0
Perceived Ideology (%)				
Very liberal	54.2	50.0	29.2	16.7
Somewhat liberal	33.3	42.3	50.0	62.5
Somewhat conservative	0	4.17	4.17	0
Very conservative	0	0	0	0
No political ideology	8.33	7.69	16.7	20.8
Agreement with Work (%)				
Strongly agree	79.2	92.3	83.3	75.0
Somewhat agree	20.8	7.69	16.7	20.8
Somewhat disagree	0	0	0	4.17
Strongly disagree	0	0	0	0
Perceived Effectiveness (%)				
Very effective	54.2	46.2	50.0	33.3
Somewhat effective	45.8	53.8	50.0	66.7
Not very effective	0	0	0	0
Not effective at all	0	0	0	0

Survey First Data Collection

NEW YORK UNIVERSITY

Mturk ID

For some reason, we were unable to capture your MTurk ID. Please, enter it below for payment purposes.

Demographics

How old are you?

Are you...?

Male

Female

Other

Which of the following best describes you? Check all that apply.

White

Black or African American

American Indian or Alaska Native

Asian

Native Hawaiian or Pacific Islander

Are you Hispanic or Latino/a?

Yes

No

How many children do you have? Include step children.

0

1

2

3

4

5 or more

How many people live in your household?

1

2

3

4

5

6 or more

Intro

In the next two pages we will ask some questions about some contemporary issues that impact Americans today. Please, answer the questions based on your personal beliefs.

Symbolic Racism Scale

Please answer the following questions according to your personal beliefs.

It’s really a matter of some people not trying hard enough; if Black people would only try harder they could be just as well off as White people.

Strongly agree

Agree

Neither agree nor disagree

Disagree

Strongly disagree

Irish, Italian, Jewish, and many other minorities overcame prejudice and worked their way up. Black people should do the same.

Strongly agree

Agree

Neither agree nor disagree

Disagree

Strongly disagree

Black people work just as hard to get ahead as most other Americans.

Strongly agree

Agree

Neither agree nor disagree

Disagree

Strongly disagree

How responsible, in general, do you hold Black people in this country for their outcomes in life?

Very responsible

Somewhat responsible

A little responsible

Not at all responsible

Black people are demanding too much from the rest of society.

Strongly agree

Agree

Neither agree nor disagree

Disagree

Strongly disagree

Some say that Black leaders have been trying to push too fast. Others feel that they haven’t pushed fast enough. What do you think?

Pushing way too fast

Pushing a bit fast

Pushing at the right pace

Pushing a bit slow

Pushing way to slowly

How much of the racial tension that exists in the United States today do you think Black people are responsible for creating?

All of the racial tension

Most of the racial tension

About half of the racial tension

A little of the racial tension

None of the racial tension

Black people generally do not complain as much as they should about their situation in society.

Strongly agree

Agree

Neither agree nor disagree

Disagree

Strongly disagree

Generations of slavery and discrimination have created conditions that make it difficult for Black people to work their way out of the lower class.

Strongly agree

Agree

Neither agree nor disagree

Disagree

Strongly disagree

Discrimination against Black people is no longer a problem in the United States.

Strongly agree

Agree

Neither agree not disagree

Disagree

Strongly disagree

Has there been a lot of real change in the position of Black people in the past few years, only some, not much at all?

A lot of positive change

A little positive change

No change

A little negative change

A lot of negative change

These page timer metrics will not be displayed to the recipient.
First Click: *0 seconds*
Last Click: *0 seconds*
Page Submit: *0 seconds*
Click Count: *0 clicks*

Environmental Concern Scale

Please answer the following questions according to your personal beliefs.

How concerned are you about environmental issues?

Not concerned at all

Somewhat unconcerned

Somewhat concerned

Very concerned

How willing would you be to pay much higher prices to help the environment?

Not at all willing

Somewhat willing

Mostly willing

Very willing

How willing would you be to accept cuts in your standard of living in order to protect the environment?

Not at all willing

Somewhat willing

Mostly willing

Very willing

In general, how dangerous do you think air pollution caused by cars is to people?

Not at all dangerous

Slightly dangerous

Somewhat dangerous

Very dangerous

Do you think air pollution caused by industry is dangerous for the environment?

Not at all dangerous

Slightly dangerous

Somewhat dangerous

Very dangerous

Many environmental threats are exaggerated.

Strongly agree

Agree

Disagree

Strongly disagree

The Earth simply cannot continue to support population growth at its present rate.

Strongly agree

Agree

Disagree

Strongly disagree

Air pollution is an issue in the area where you live.

Strongly agree

Agree

Disagree

Strongly disagree

These page timer metrics will not be displayed to the recipient.
First Click: *0 seconds*
Last Click: *0 seconds*
Page Submit: *0 seconds*
Click Count: *0 clicks*

Risk - White Americans

White Americans' lives are negatively impacted by environmental issues.

Strongly agree

Agree

Disagree

Strongly disagree

Risk - Black Americans

Black Americans' lives are negatively impacted by environmental issues.

Strongly agree

Agree

Disagree

Strongly disagree

Risk - Poor Americans

Poor Americans' lives are negatively impacted by environmental issues.

Strongly agree

Agree

Disagree

Strongly disagree

Risk - Self

Your own life is negatively impacted by environmental issues.

Strongly agree

Agree

Disagree

Strongly disagree

Political Ideology

Where would you place yourself on this scale?

Extremely liberal

Liberal

Slightly liberal

Moderate; middle of the road

Slightly conservative

Conservative

Extremely conservative

Prefer not to say

If you had to choose, would you consider yourself more liberal or conservative?

Liberal

Conservative

Party Concern about Enviro

How concerned do you believe most conservatives are about the environment?

Not concerned at all

Somewhat unconcerned

Somewhat concerned

Very concerned

How concerned do you believe most liberals are about the environment?

Not concerned at all

Somewhat unconcerned

Somewhat concerned

Very concerned

Party Concern about Racism

How concerned do you believe most conservatives are about discrimination on the basis of race?

Not concerned at all

Somewhat unconcerned

Somewhat concerned

Very concerned

How concerned do you believe most liberals are about discrimination on the basis of race?

Not concerned at all

Somewhat unconcerned

Somewhat concerned

Very concerned

Demographic Questions

What is the highest education level you have completed?

No high school degree
High school degree or equivalent (ie. GED)
Some college, no degree
Associate degree (ie. AA, AS)
Bachelors degree (ie. BA, BS)
Master's degree (ie. MA, MS)
Professional degree (ie. MD, DDS, DVM)
Doctorate (ie. PhD, EdD)

At the end of January 2020 what best described your employment situation?

Full-time employed
Part-time employed
Self-employed
Unemployed
Caregiver or homemaker
Retired
Full-time student

What best describes your current employment situation?

Full-time employed
Part-time employed
Self-employed
Unemployed
Caregiver or homemaker
Retired
Full-time student

Do you presently have any kind of health insurance?

Yes
No
Not sure

Would you say that in general your health is...

Excellent

Very good

Good

Fair

Poor

What was your household income for the year 2019 before taxes?

Less than $10,000

$10,000-$24,999

$25,000-$49,999

$50,000-$74,999

$75,000-$99,999

$100,000-$150,000

More than $150,000

Prefer not to answer

In which zip code are you currently residing in? We will use this information for statistical purposes only.

Powered by Qualtrics

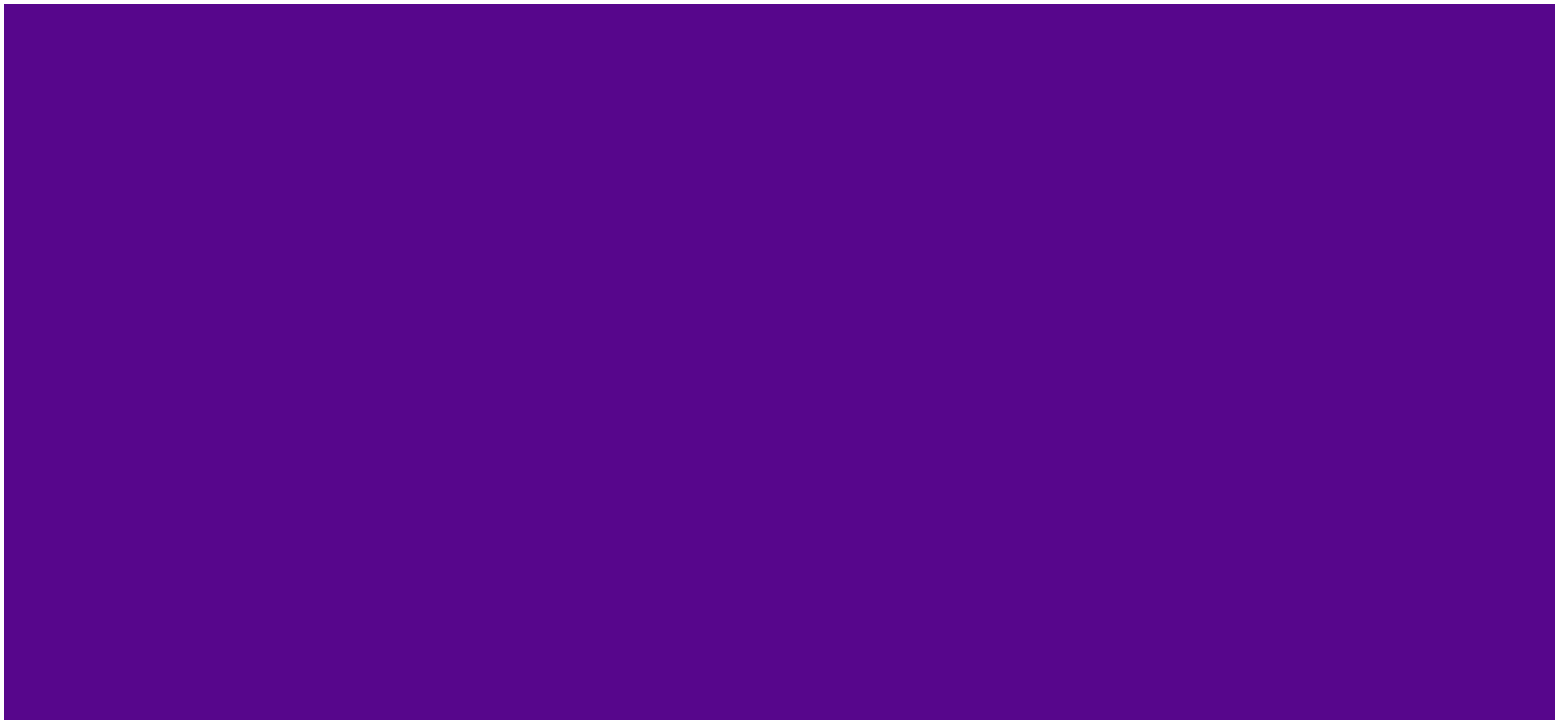

Survey Second Data Collection

MTurk ID

For some reason, we were unable to capture your MTurk ID. Please, enter it below for payment purposes.

Intro

The following page contains a short text. Please read it and answer the questions that follow.

Liberal 1

Please read the following passage about the risks posed by air pollution. On the following page you will be asked a short set of questions to verify your understanding. In order to earn your bonus of $0.50, you must answer the questions correctly on the first try.

A recent national assessment found that air pollution is responsible for elevated lifetime cancer risks nationwide. These effects were found to be even more severe in highly polluted areas, where nearly 14 million people have lifetime cancer risks greater than 100 in a million. Health risks are highest in populous areas near multiple emission sources, such as industrial facilities, large roadways or transportation facilities. The impact of air pollution is not limited to health, but also impacts our economy in lost productivity. Ozone smog is associated with 750,000 summertime asthma attacks in children and 500,000 missed school days. Among adults, pollution results in 2,000 asthma related emergency room visits, 600 hospital admissions, and 1.5 million reduced activity days.

These numbers are not merely statistics, but real people's lives. For the Washingtons, like for most American families, air pollution was not a daily

concern. That changed when their 4 year old daughter, Tyra, had her first asthma attack while playing at the playground close to where they lived in the Chicago area. As an energetic little girl, the swings and monkey bars had always been her favorite. After her diagnosis, it became difficult for her parents to keep her active and entertained, especially given their busy schedules as they both work in sales. A year later, their 11 year old son, Terell, developed asthma as well, preventing him from playing soccer, which he had always looked forward to after school. They soon realized that many of their neighbors were experiencing similar problems. Their story is just one example of the air pollution related health repercussions that are becoming increasingly prevalent.

Policies that reduce pollution can help protect the health of American people and reduce the gaping health inequities that threaten the wellbeing of communities across the country. Taking action on pollution not only protects the quality of our air, but also safeguards the health and livelihood of vulnerable Americans, including those unable to afford a healthier living space. Protecting the environment is a matter of justice, care, and equity. The men and women barely able to make ends meet should not have to struggle to breathe. Their wellbeing and equal rights will not be possible if we continue to pollute the world around us. The bottom line is that fighting against air pollution is fighting to uphold equality and ensuring that the health of marginalized communities is not put at risk by corporate greed and the 1%.

These page timer metrics will not be displayed to the recipient.
First Click: *0 seconds*
Last Click: *0 seconds*
Page Submit: *0 seconds*
Click Count: *0 clicks*

Liberal 2

Please read the following passage about the risks posed by air pollution. On the following page you will be asked a short set of questions to verify your understanding. In order to earn your bonus of $0.50, you must answer the questions correctly on the first try.

A recent national assessment found that air pollution is responsible for elevated lifetime cancer risks nationwide. These effects were found to be even more severe in highly polluted areas, where nearly 14 million people have lifetime cancer risks greater than 100 in a million. Health risks are highest in populous areas near multiple emission sources, such as industrial facilities, large roadways or transportation facilities. The impact of air pollution is not limited to health, but also impacts our economy in lost productivity. Ozone smog is associated with 750,000 summertime asthma attacks in children and 500,000 missed school days. Among adults, pollution results in 2,000 asthma related emergency room visits, 600 hospital admissions, and 1.5 million reduced activity days.

These numbers are not merely statistics, but real people’s lives. For the Meyers, like for most American families, air pollution was not a daily concern. That changed when their 4 year old daughter, Amy, had her first asthma attack while playing at the playground close to where they lived in the Chicago area. As an energetic little girl, the swings and monkey bars had always been her favorite. After her diagnosis, it became difficult for her parents to keep her active and entertained, especially given their busy schedules as they both work in sales. A year later, their 11 year old son, Cody, developed asthma as well, preventing him from playing soccer, which he had always looked forward to after school. They soon realized that many of their neighbors were experiencing similar problems. Their story is just one example of the air pollution related health repercussions that are becoming increasingly prevalent.

Policies that reduce pollution can help protect the health of American people and reduce the gaping health inequities that threaten the wellbeing of communities across the country. Taking action on pollution not only protects the quality of our air, but also safeguards the health and livelihood of vulnerable Americans, including those unable to afford a healthier living space. Protecting the environment is a matter of justice, care, and equity. The men and women barely able to make ends meet should not have to struggle to breathe. Their wellbeing and equal rights will not be possible if we continue to pollute the world around us. The bottom line is that fighting against air pollution is fighting to uphold equality and ensuring that the health of marginalized communities is not put at risk by corporate greed and the 1%.

These page timer metrics will not be displayed to the recipient.
First Click: *0 seconds*
Last Click: *0 seconds*
Page Submit: *0 seconds*
Click Count: *0 clicks*

Conservative 1

Please read the following passage about the risks posed by air pollution. On the following page you will be asked a short set of questions to verify your understanding. In order to earn your bonus of $0.50, you must answer the questions correctly on the first try.

A recent national assessment found that air pollution is responsible for elevated lifetime cancer risks nationwide. These effects were found to be even more severe in highly polluted areas, where nearly 14 million people have lifetime cancer risks greater than 100 in a million. Health risks are highest in populous areas near multiple emission sources, such as industrial facilities, large roadways or transportation facilities. The impact of air pollution is not limited to health, but also impacts our economy in lost productivity. Ozone smog is associated with 750,000 summertime asthma attacks in children and 500,000 missed school days. Among adults, pollution results in 2,000 asthma related emergency room visits, 600 hospital admissions, and 1.5 million reduced activity days.

These numbers are not merely statistics, but real people's lives. For the Washingtons, like for most American families, air pollution was not a daily concern. That changed when their 4 year old daughter, Tyra, had her first asthma attack while playing at the playground close to where they lived in the Chicago area. As an energetic little girl, the swings and monkey bars had always been her favorite. After her diagnosis, it became difficult for her parents to keep her active and entertained, especially given their busy schedules as they both work in sales. A year later, their 11 year old son, Terell, developed asthma as well, preventing him from playing soccer, which he had always looked forward to after school. They soon realized that many of their neighbors were experiencing

similar problems. Their story is just one example of the air pollution related health repercussions that are becoming increasingly prevalent.

Policies that reduce pollution can help protect the purity of American air and eliminate the toxins and particulate matter that threatens to clog the lungs of Americans across the country. Taking action on pollution not only protects the quality of our air, but also safeguards the jobs and businesses of hardworking Americans, including those unable to work due to their health. Protecting the environment is a matter of honor, patriotism and respect. The men and women working hard to contribute to our great nation should not have to struggle to breathe. Their dignity and prosperity will not be possible if we continue to pollute the world around us. The bottom line is that fighting against air pollution is fighting to uphold the constitutional rights of American citizens and ensuring that our robust economy is not weakened by unnecessary health risks.

These page timer metrics will not be displayed to the recipient.
First Click: *0 seconds*
Last Click: *0 seconds*
Page Submit: *0 seconds*
Click Count: *0 clicks*

Conservative 2

Please read the following passage about the risks posed by air pollution. On the following page you will be asked a short set of questions to verify your understanding. In order to earn your bonus of $0.50, you must answer the questions correctly on the first try.

A recent national assessment found that air pollution is responsible for elevated lifetime cancer risks nationwide. These effects were found to be even more severe in highly polluted areas, where nearly 14 million people have lifetime cancer risks greater than 100 in a million. Health risks are highest in populous areas near multiple emission sources, such as industrial facilities, large roadways or transportation facilities. The impact of air pollution is not limited to health, but

also impacts our economy in lost productivity. Ozone smog is associated with 750,000 summertime asthma attacks in children and 500,000 missed school days. Among adults, pollution results in 2,000 asthma related emergency room visits, 600 hospital admissions, and 1.5 million reduced activity days.

These numbers are not merely statistics, but real people's lives. For the Meyers, like for most American families, air pollution was not a daily concern. That changed when their 4 year old daughter, Amy, had her first asthma attack while playing at the playground close to where they lived in the Chicago area. As an energetic little girl, the swings and monkey bars had always been her favorite. After her diagnosis, it became difficult for her parents to keep her active and entertained, especially given their busy schedules as they both work in sales. A year later, their 11 year old son, Cody, developed asthma as well, preventing him from playing soccer, which he had always looked forward to after school. They soon realized that many of their neighbors were experiencing similar problems. Their story is just one example of the air pollution related health repercussions that are becoming increasingly prevalent.

Policies that reduce pollution can help protect the purity of American air and eliminate the toxins and particulate matter that threatens to clog the lungs of Americans across the country. Taking action on pollution not only protects the quality of our air, but also safeguards the jobs and businesses of hardworking Americans, including those unable to work due to their health. Protecting the environment is a matter of honor, patriotism and respect. The men and women working hard to contribute to our great nation should not have to struggle to breathe. Their dignity and prosperity will not be possible if we continue to pollute the world around us. The bottom line is that fighting against air pollution is fighting to uphold the constitutional rights of American citizens and ensuring that our robust economy is not weakened by unnecessary health risks.

These page timer metrics will not be displayed to the recipient.
First Click: *0 seconds*
Last Click: *0 seconds*
Page Submit: *0 seconds*
Click Count: *0 clicks*

Comprehension Check

Answer the following questions based on the passage you just read. You may scroll down to reference the passage again.

What was the text you just read about?

Floods

Fires

Air pollution

Water pollution

What health risks were mentioned in the text you read?

Asthma and cancer

Asthma and diabetes

Cancer and diabetes

Sexually transmitted diseases

What other risks did the text mention?

Deforestation

Mental health

COVID-19

Lost productivity

According to the passage, policies against air pollution would protect the health and livelihood of vulnerable Americans.

True

False

According to the passage, policies against air pollution would protect the jobs and businesses of hardworking Americans.

True

False

A recent national assessment found that air pollution is responsible for elevated lifetime cancer risks nationwide. These effects were found to be even more severe in highly polluted areas, where nearly 14 million people have lifetime cancer risks greater than 100 in a million. Health risks are highest in populous areas near multiple emission sources, such as industrial facilities, large roadways or transportation facilities. The impact of air pollution is not limited to health, but also impacts our economy in lost productivity. Ozone smog is associated with 750,000 summertime asthma attacks in children and 500,000 missed school days. Among adults, pollution results in 2,000 asthma related emergency room visits, 600 hospital admissions, and 1.5 million reduced activity days.

These numbers are not merely statistics, but real people's lives. For the Washingtons, like for most American families, air pollution was not a daily concern. That changed when their 4 year old daughter, Tyra, had her first asthma attack while playing at the playground close to where they lived in the Chicago area. As an energetic little girl, the swings and monkey bars had always been her favorite. After her diagnosis, it became difficult for her parents to keep her active and entertained, especially given their busy schedules as they both work in sales. A year later, their 11 year old son, Terell, developed asthma as well, preventing him from playing soccer, which he had always looked forward to after school. They soon realized that many of their neighbors were experiencing similar problems. Their story is just one example of the air pollution related health repercussions that are becoming increasingly prevalent.

Policies that reduce pollution can help protect the health of American people and reduce the gaping health inequities that threaten the wellbeing of communities across the country. Taking action on pollution not only protects the quality of our air, but also safeguards the health and livelihood of vulnerable Americans, including those unable to afford a healthier living space. Protecting the environment is a matter of justice, care, and equity. The men and women barely able to make ends meet should not have to struggle to breathe. Their wellbeing and equal rights will not be possible if we continue to pollute the world around us. The bottom line is that fighting against air pollution is fighting to uphold equality and ensuring that the health of marginalized communities is not put at risk by corporate greed and the 1%.

A recent national assessment found that air pollution is responsible for elevated lifetime cancer risks nationwide. These effects were found to be even more severe in highly polluted areas, where nearly 14 million people have lifetime cancer risks greater than 100 in a million. Health risks are highest in populous areas near multiple emission sources, such as industrial facilities, large roadways or transportation facilities. The impact of air pollution is not limited to health, but also impacts our economy in lost productivity. Ozone smog is associated with 750,000 summertime asthma attacks in children and 500,000 missed school days. Among adults, pollution results in 2,000 asthma related emergency room visits, 600 hospital admissions, and 1.5 million reduced activity days.

These numbers are not merely statistics, but real people's lives. For the Washingtons, like for most American families, air pollution was not a daily concern. That changed when their 4 year old daughter, Tyra, had her first asthma attack while playing at the playground close to where they lived in the Chicago area. As an energetic little girl, the swings and monkey bars had always been her favorite. After her diagnosis, it became difficult for her parents to keep her active and entertained, especially given their busy schedules as they both work in sales. A year later, their 11 year old son, Terell, developed asthma as well, preventing him from playing soccer, which he had always looked forward to after school. They soon realized that many of their neighbors were experiencing similar problems. Their story is just one example of the air pollution related health repercussions that are becoming increasingly prevalent.

Policies that reduce pollution can help protect the purity of American air and eliminate the toxins and particulate matter that threatens to clog the lungs of Americans across the country. Taking action on pollution not only protects the quality of our air, but also safeguards the jobs and businesses of hardworking Americans, including those unable to work due to their health. Protecting the environment is a matter of honor, patriotism and respect. The men and women working hard to contribute to our great nation should not have to struggle to breathe. Their dignity and prosperity will not be possible if we continue to pollute the world around us. The bottom line is that fighting against air pollution is fighting to uphold the constitutional rights of American citizens and ensuring that our robust economy is not weakened by unnecessary health risks.

A recent national assessment found that air pollution is responsible for elevated lifetime cancer risks nationwide. These effects were found to be even more severe in highly polluted areas, where nearly 14 million people have lifetime cancer risks greater than 100 in a million. Health risks are highest in populous areas near multiple emission sources, such as industrial facilities, large roadways or transportation facilities. The impact of air pollution is not limited to health, but also impacts our economy in lost productivity. Ozone smog is associated with 750,000 summertime asthma attacks in children and 500,000 missed school days. Among adults, pollution results in 2,000 asthma related emergency room visits, 600 hospital admissions, and 1.5 million reduced activity days.

These numbers are not merely statistics, but real people's lives. For the Meyers, like for most American families, air pollution was not a daily concern. That changed when their 4 year old daughter, Amy, had her first asthma attack while playing at the playground close to where they lived in the Chicago area. As an energetic little girl, the swings and monkey bars had always been her favorite. After her diagnosis, it became difficult for her parents to keep her active and entertained, especially given their busy schedules as they both work in sales. A year later, their 11 year old son, Cody, developed asthma as well, preventing him from playing soccer, which he had always looked forward to after school. They soon realized that many of their neighbors were experiencing similar problems. Their story is just one example of the air pollution related health repercussions that are becoming increasingly prevalent.

Policies that reduce pollution can help protect the health of American people and reduce the gaping health inequities that threaten the wellbeing of communities across the country. Taking action on pollution not only protects the quality of our air, but also safeguards the health and livelihood of vulnerable Americans, including those unable to afford a healthier living space. Protecting the environment is a matter of justice, care, and equity. The men and women barely able to make ends meet should not have to struggle to breathe. Their wellbeing and equal rights will not be possible if we continue to pollute the world around us. The bottom line is that fighting against air pollution is fighting to uphold equality and ensuring that the health of marginalized communities is not put at risk by corporate greed and the 1%.

A recent national assessment found that air pollution is responsible for elevated lifetime cancer risks nationwide. These effects were found to be even more severe in highly polluted areas, where nearly 14 million people have lifetime cancer risks greater than 100 in a million. Health risks are highest in populous areas near multiple emission sources, such as industrial facilities, large roadways or transportation facilities. The impact of air pollution is not limited to health, but also impacts our economy in lost productivity. Ozone smog is associated with 750,000 summertime asthma attacks in children and 500,000 missed school days. Among adults, pollution results in 2,000 asthma related emergency room visits, 600 hospital admissions, and 1.5 million reduced activity days.

These numbers are not merely statistics, but real people's lives. For the Meyers, like for most American families, air pollution was not a daily concern. That changed when their 4 year old daughter, Amy, had her first asthma attack while playing at the playground close to where they lived in the Chicago area. As an energetic little girl, the swings and monkey bars had always been her favorite. After her diagnosis, it became difficult for her parents to keep her active and entertained, especially given their busy schedules as they both work in sales. A year later, their 11 year old son, Cody, developed asthma as well, preventing him from playing soccer, which he had always looked forward to after school. They soon realized that many of their neighbors were experiencing similar problems. Their story is just one example of the air pollution related health repercussions that are becoming increasingly prevalent.

Policies that reduce pollution can help protect the purity of American air and eliminate the toxins and particulate matter that threatens to clog the lungs of Americans across the country. Taking action on pollution not only protects the quality of our air, but also safeguards the jobs and businesses of hardworking Americans, including those unable to work due to their health. Protecting the environment is a matter of honor, patriotism and respect. The men and women working hard to contribute to our great nation should not have to struggle to breathe. Their dignity and prosperity will not be possible if we continue to pollute the world around us. The bottom line is that fighting against air pollution is fighting to uphold the constitutional rights of American citizens and ensuring that our robust economy is not weakened by unnecessary health risks.

Correct Notification

You have answered all questions correctly! Please continue.

Action Opportunity

You have now received 10 virtual points. You will have the opportunity to donate some, all, or none of your points towards an organization combatting air pollution, and keep the rest.

The points that you keep, will each be converted into a ticket to enter a raffle for two $100 Amazon gift cards, which will be awarded to participants in this study. To understand how tickets work, consider the following example: if participant A keeps 5 tickets, while participant B keeps 10, Participant B has twice the chances to be selected for the gift cards.

Each point you give corresponds to a $0.50 donation towards EarthJustice, an organization of lawyers working to preserve our air, land, and water. For example, if you keep 7 tickets, and donate 3, EarthJustice will receive $1.50 on your behalf.

We invite you to read the quoted passage from earthjustice.org below as you consider making a donation of points. To read more about Earthjustice and their Clean Air focus area, click here.

"Our legal work has saved irreplaceable wildlands, cleaned up the air we breathe, and fueled the rise of 100% clean energy. It has protected countless species on the brink of extinction, and secured long-overdue, historic limits on our nation’s worst polluting industries. When we go to court, we get results. And we’re able to do this with the generous support of people like you."

The bottom line is that fighting against air pollution is fighting to uphold equality and ensuring that the health of marginalized communities is not put at risk by corporate greed and the 1%, a fight that would improve the wellbeing of families like the Washingtons.

The bottom line is that fighting against air pollution is fighting to uphold equality and ensuring that the health of marginalized communities is not put at risk by corporate greed and the 1%, a fight that would improve the wellbeing of families like the Meyers.

The bottom line is that fighting against air pollution is fighting to uphold the constitutional rights of American citizens and ensuring that our robust economy is not weakened by unnecessary health risks, a fight that would improve the wellbeing of families like the Washingtons.

The bottom line is that fighting against air pollution is fighting to uphold the constitutional rights of American citizens and ensuring that our robust economy is not weakened by unnecessary health risks, a fight that would improve the wellbeing of families like the Meyers.

Please enter the number of points you would like to donate.

Of the 10 points I have been awarded,

I would like to donate ______.

And I would like to keep

EarthJustice Perceptions

How effective do you think EarthJustice is in their work compared to other organizations?

Very effective

Somewhat effective

Not very effective

Not at all effective

How would you characterize EarthJustice's ideology?

Very liberal

Somewhat liberal

Somewhat conservative

Very conservative

They do not appear to have a political ideology

Race and Risk Perception

What race or ethnicity do you associate with the family mentioned in the passage?

White

Black

Hispanic or Latino

Other

None

After reading this passage, how serious of a problem do you consider air pollution to be?

Not at all serious

Slightly serious

Somewhat serious

Very serious

A recent national assessment found that air pollution is responsible for elevated lifetime cancer risks nationwide. These effects were found to be even more severe in highly polluted areas, where nearly 14 million people have lifetime cancer risks greater than 100 in a million. Health risks are highest in populous areas near multiple emission sources, such as industrial facilities, large roadways or transportation facilities. The impact of air pollution is not limited to health, but also impacts our economy in lost productivity. Ozone smog is associated with 750,000 summertime asthma attacks in children and 500,000 missed school days. Among adults, pollution results in 2,000 asthma related emergency room visits, 600 hospital admissions, and 1.5 million reduced activity days.

These numbers are not merely statistics, but real people's lives. For the Washingtons, like for most American families, air pollution was not a daily concern. That changed when their 4 year old daughter, Tyra, had her first asthma attack while playing at the playground close to where they lived in the Chicago area. As an energetic little girl, the swings and monkey bars had always been her favorite. After her diagnosis, it became difficult for her parents to keep her active and entertained, especially given their busy schedules as they both work in sales. A year later, their 11 year old son, Terell, developed asthma as well, preventing him from playing soccer, which he had always looked forward to after school. They soon realized that many of their neighbors were experiencing similar problems. Their story is just one example of the air pollution related health repercussions that are becoming increasingly prevalent.

Policies that reduce pollution can help protect the health of American people and reduce the gaping health inequities that threaten the wellbeing of communities across the country. Taking action on pollution not only protects the quality of our air, but also safeguards the health and livelihood of vulnerable Americans, including those unable to afford a healthier living space. Protecting the environment is a matter of justice, care, and equity. The men and women barely able to make ends meet should not have to struggle to breathe. Their wellbeing and equal rights will not be possible if we continue to pollute the world around us. The bottom line is that fighting against air pollution is fighting to uphold equality and ensuring that the health of marginalized communities is not put at risk by corporate greed and the 1%.

A recent national assessment found that air pollution is responsible for elevated lifetime cancer risks nationwide. These effects were found to be even more severe in highly polluted areas, where nearly 14 million people have lifetime cancer risks greater than 100 in a million. Health risks are highest in populous areas near multiple emission sources, such as industrial facilities, large roadways or transportation facilities. The impact of air pollution is not limited to health, but also impacts our economy in lost productivity. Ozone smog is associated with 750,000 summertime asthma attacks in children and 500,000 missed school days. Among adults, pollution results in 2,000 asthma related emergency room visits, 600 hospital admissions, and 1.5 million reduced activity days.

These numbers are not merely statistics, but real people's lives. For the Meyers, like for most American families, air pollution was not a daily concern. That changed when their 4 year old daughter, Amy, had her first asthma attack while playing at the playground close to where they lived in the Chicago area. As an energetic little girl, the swings and monkey bars had always been her favorite. After her diagnosis, it became difficult for her parents to keep her active and entertained, especially given their busy schedules as they both work in sales. A year later, their 11 year old son, Cody, developed asthma as well, preventing him from playing soccer, which he had always looked forward to after school. They soon realized that many of their neighbors were experiencing similar problems. Their story is just one example of the air pollution related health repercussions that are becoming increasingly prevalent.

Policies that reduce pollution can help protect the health of American people and reduce the gaping health inequities that threaten the wellbeing of communities across the country. Taking action on pollution not only protects the quality of our air, but also safeguards the health and livelihood of vulnerable Americans, including those unable to afford a healthier living space. Protecting the environment is a matter of justice, care, and equity. The men and women barely able to make ends meet should not have to struggle to breathe. Their wellbeing and equal rights will not be possible if we continue to pollute the world around us. The bottom line is that fighting against air pollution is fighting to uphold equality and ensuring that the health of marginalized communities is not put at risk by corporate greed and the 1%.

A recent national assessment found that air pollution is responsible for elevated lifetime cancer risks nationwide. These effects were found to be even more severe in highly polluted areas, where nearly 14 million people have lifetime cancer risks greater than 100 in a million. Health risks are highest in populous areas near multiple emission sources, such as industrial facilities, large roadways or transportation facilities. The impact of air pollution is not limited to health, but also impacts our economy in lost productivity. Ozone smog is associated with 750,000 summertime asthma attacks in children and 500,000 missed school

days. Among adults, pollution results in 2,000 asthma related emergency room visits, 600 hospital admissions, and 1.5 million reduced activity days.

These numbers are not merely statistics, but real people's lives. For the Washingtons, like for most American families, air pollution was not a daily concern. That changed when their 4 year old daughter, Tyra, had her first asthma attack while playing at the playground close to where they lived in the Chicago area. As an energetic little girl, the swings and monkey bars had always been her favorite. After her diagnosis, it became difficult for her parents to keep her active and entertained, especially given their busy schedules as they both work in sales. A year later, their 11 year old son, Terell, developed asthma as well, preventing him from playing soccer, which he had always looked forward to after school. They soon realized that many of their neighbors were experiencing similar problems. Their story is just one example of the air pollution related health repercussions that are becoming increasingly prevalent.

Policies that reduce pollution can help protect the purity of American air and eliminate the toxins and particulate matter that threatens to clog the lungs of Americans across the country. Taking action on pollution not only protects the quality of our air, but also safeguards the jobs and businesses of hardworking Americans, including those unable to work due to their health. Protecting the environment is a matter of honor, patriotism and respect. The men and women working hard to contribute to our great nation should not have to struggle to breathe. Their dignity and prosperity will not be possible if we continue to pollute the world around us. The bottom line is that fighting against air pollution is fighting to uphold the constitutional rights of American citizens and ensuring that our robust economy is not weakened by unnecessary health risks.

A recent national assessment found that air pollution is responsible for elevated lifetime cancer risks nationwide. These effects were found to be even more severe in highly polluted areas, where nearly 14 million people have lifetime cancer risks greater than 100 in a million. Health risks are highest in populous areas near multiple emission sources, such as industrial facilities, large roadways or transportation facilities. The impact of air pollution is not limited to health, but also impacts our economy in lost productivity. Ozone smog is associated with

750,000 summertime asthma attacks in children and 500,000 missed school days. Among adults, pollution results in 2,000 asthma related emergency room visits, 600 hospital admissions, and 1.5 million reduced activity days.

These numbers are not merely statistics, but real people's lives. For the Meyers, like for most American families, air pollution was not a daily concern. That changed when their 4 year old daughter, Amy, had her first asthma attack while playing at the playground close to where they lived in the Chicago area. As an energetic little girl, the swings and monkey bars had always been her favorite. After her diagnosis, it became difficult for her parents to keep her active and entertained, especially given their busy schedules as they both work in sales. A year later, their 11 year old son, Cody, developed asthma as well, preventing him from playing soccer, which he had always looked forward to after school. They soon realized that many of their neighbors were experiencing similar problems. Their story is just one example of the air pollution related health repercussions that are becoming increasingly prevalent.

Policies that reduce pollution can help protect the purity of American air and eliminate the toxins and particulate matter that threatens to clog the lungs of Americans across the country. Taking action on pollution not only protects the quality of our air, but also safeguards the jobs and businesses of hardworking Americans, including those unable to work due to their health. Protecting the environment is a matter of honor, patriotism and respect. The men and women working hard to contribute to our great nation should not have to struggle to breathe. Their dignity and prosperity will not be possible if we continue to pollute the world around us. The bottom line is that fighting against air pollution is fighting to uphold the constitutional rights of American citizens and ensuring that our robust economy is not weakened by unnecessary health risks.

Follow Up Questions

On the following scales, please indicate your impression of the passage you read.

The passage seemed...

...not biased at all ...very biased

1 2 3 4 5 6 7

The facts about the impact of air pollution on health and productivity seemed...

...not credible at all ...very credible

1 2 3 4 5 6 7

The passage seemed to lean politically...

...liberal ...conservative

1 2 3 4 5 6 7

The tone of the passage was...

...very negative ...very positive

1 2 3 4 5 6 7

The passage makes me feel that...

...change is impossible ...change is definately possible

1 2 3 4 5 6 7

A recent national assessment found that air pollution is responsible for elevated lifetime cancer risks nationwide. These effects were found to be even more severe in highly polluted areas, where nearly 14 million people have lifetime cancer risks greater than 100 in a million. Health risks are highest in populous areas near multiple emission sources, such as industrial facilities, large roadways or transportation facilities. The impact of air pollution is not limited to health, but also impacts our economy in lost productivity. Ozone smog is associated with 750,000 summertime asthma attacks in children and 500,000 missed school

days. Among adults, pollution results in 2,000 asthma related emergency room visits, 600 hospital admissions, and 1.5 million reduced activity days.

These numbers are not merely statistics, but real people's lives. For the Washingtons, like for most American families, air pollution was not a daily concern. That changed when their 4 year old daughter, Tyra, had her first asthma attack while playing at the playground close to where they lived in the Chicago area. As an energetic little girl, the swings and monkey bars had always been her favorite. After her diagnosis, it became difficult for her parents to keep her active and entertained, especially given their busy schedules as they both work in sales. A year later, their 11 year old son, Terell, developed asthma as well, preventing him from playing soccer, which he had always looked forward to after school. They soon realized that many of their neighbors were experiencing similar problems. Their story is just one example of the air pollution related health repercussions that are becoming increasingly prevalent.

Policies that reduce pollution can help protect the health of American people and reduce the gaping health inequities that threaten the wellbeing of communities across the country. Taking action on pollution not only protects the quality of our air, but also safeguards the health and livelihood of vulnerable Americans, including those unable to afford a healthier living space. Protecting the environment is a matter of justice, care, and equity. The men and women barely able to make ends meet should not have to struggle to breathe. Their wellbeing and equal rights will not be possible if we continue to pollute the world around us. The bottom line is that fighting against air pollution is fighting to uphold equality and ensuring that the health of marginalized communities is not put at risk by corporate greed and the 1%.

A recent national assessment found that air pollution is responsible for elevated lifetime cancer risks nationwide. These effects were found to be even more severe in highly polluted areas, where nearly 14 million people have lifetime cancer risks greater than 100 in a million. Health risks are highest in populous areas near multiple emission sources, such as industrial facilities, large roadways or transportation facilities. The impact of air pollution is not limited to health, but also impacts our economy in lost productivity. Ozone smog is associated with

750,000 summertime asthma attacks in children and 500,000 missed school days. Among adults, pollution results in 2,000 asthma related emergency room visits, 600 hospital admissions, and 1.5 million reduced activity days.

These numbers are not merely statistics, but real people's lives. For the Meyers, like for most American families, air pollution was not a daily concern. That changed when their 4 year old daughter, Amy, had her first asthma attack while playing at the playground close to where they lived in the Chicago area. As an energetic little girl, the swings and monkey bars had always been her favorite. After her diagnosis, it became difficult for her parents to keep her active and entertained, especially given their busy schedules as they both work in sales. A year later, their 11 year old son, Cody, developed asthma as well, preventing him from playing soccer, which he had always looked forward to after school. They soon realized that many of their neighbors were experiencing similar problems. Their story is just one example of the air pollution related health repercussions that are becoming increasingly prevalent.

Policies that reduce pollution can help protect the health of American people and reduce the gaping health inequities that threaten the wellbeing of communities across the country. Taking action on pollution not only protects the quality of our air, but also safeguards the health and livelihood of vulnerable Americans, including those unable to afford a healthier living space. Protecting the environment is a matter of justice, care, and equity. The men and women barely able to make ends meet should not have to struggle to breathe. Their wellbeing and equal rights will not be possible if we continue to pollute the world around us. The bottom line is that fighting against air pollution is fighting to uphold equality and ensuring that the health of marginalized communities is not put at risk by corporate greed and the 1%.

A recent national assessment found that air pollution is responsible for elevated lifetime cancer risks nationwide. These effects were found to be even more severe in highly polluted areas, where nearly 14 million people have lifetime cancer risks greater than 100 in a million. Health risks are highest in populous areas near multiple emission sources, such as industrial facilities, large roadways or transportation facilities. The impact of air pollution is not limited to health, but

also impacts our economy in lost productivity. Ozone smog is associated with 750,000 summertime asthma attacks in children and 500,000 missed school days. Among adults, pollution results in 2,000 asthma related emergency room visits, 600 hospital admissions, and 1.5 million reduced activity days.

These numbers are not merely statistics, but real people's lives. For the Washingtons, like for most American families, air pollution was not a daily concern. That changed when their 4 year old daughter, Tyra, had her first asthma attack while playing at the playground close to where they lived in the Chicago area. As an energetic little girl, the swings and monkey bars had always been her favorite. After her diagnosis, it became difficult for her parents to keep her active and entertained, especially given their busy schedules as they both work in sales. A year later, their 11 year old son, Terell, developed asthma as well, preventing him from playing soccer, which he had always looked forward to after school. They soon realized that many of their neighbors were experiencing similar problems. Their story is just one example of the air pollution related health repercussions that are becoming increasingly prevalent.

Policies that reduce pollution can help protect the purity of American air and eliminate the toxins and particulate matter that threatens to clog the lungs of Americans across the country. Taking action on pollution not only protects the quality of our air, but also safeguards the jobs and businesses of hardworking Americans, including those unable to work due to their health. Protecting the environment is a matter of honor, patriotism and respect. The men and women working hard to contribute to our great nation should not have to struggle to breathe. Their dignity and prosperity will not be possible if we continue to pollute the world around us. The bottom line is that fighting against air pollution is fighting to uphold the constitutional rights of American citizens and ensuring that our robust economy is not weakened by unnecessary health risks.

A recent national assessment found that air pollution is responsible for elevated lifetime cancer risks nationwide. These effects were found to be even more severe in highly polluted areas, where nearly 14 million people have lifetime cancer risks greater than 100 in a million. Health risks are highest in populous areas near multiple emission sources, such as industrial facilities, large roadways

or transportation facilities. The impact of air pollution is not limited to health, but also impacts our economy in lost productivity. Ozone smog is associated with 750,000 summertime asthma attacks in children and 500,000 missed school days. Among adults, pollution results in 2,000 asthma related emergency room visits, 600 hospital admissions, and 1.5 million reduced activity days.

These numbers are not merely statistics, but real people’s lives. For the Meyers, like for most American families, air pollution was not a daily concern. That changed when their 4 year old daughter, Amy, had her first asthma attack while playing at the playground close to where they lived in the Chicago area. As an energetic little girl, the swings and monkey bars had always been her favorite. After her diagnosis, it became difficult for her parents to keep her active and entertained, especially given their busy schedules as they both work in sales. A year later, their 11 year old son, Cody, developed asthma as well, preventing him from playing soccer, which he had always looked forward to after school. They soon realized that many of their neighbors were experiencing similar problems. Their story is just one example of the air pollution related health repercussions that are becoming increasingly prevalent.

Policies that reduce pollution can help protect the purity of American air and eliminate the toxins and particulate matter that threatens to clog the lungs of Americans across the country. Taking action on pollution not only protects the quality of our air, but also safeguards the jobs and businesses of hardworking Americans, including those unable to work due to their health. Protecting the environment is a matter of honor, patriotism and respect. The men and women working hard to contribute to our great nation should not have to struggle to breathe. Their dignity and prosperity will not be possible if we continue to pollute the world around us. The bottom line is that fighting against air pollution is fighting to uphold the constitutional rights of American citizens and ensuring that our robust economy is not weakened by unnecessary health risks.

Social Distance from Risk

This passage made me feel that my family and friends are...

...not at all at risk ...very much at risk
...not at all at risk ...very much at risk

1 2 3 4 5 6 7
1 2 3 4 5 6 7

This passage made me feel that the wider community is...

...not at all at risk ...very much at risk

1 2 3 4 5 6 7

These numbers are not merely statistics, but real people’s lives. For the Washingtons, like for most American families, air pollution was not a daily concern. That changed when their 4 year old daughter, Tyra, had her first asthma attack while playing at the playground close to where they lived in the Chicago area. As an energetic little girl, the swings and monkey bars had always been her favorite. After her diagnosis, it became difficult for her parents to keep her active and entertained, especially given their busy schedules as they both work in sales. A year later, their 11 year old son, Terell, developed asthma as well, preventing him from playing soccer, which he had always looked forward to after school. They soon realized that many of their neighbors were experiencing similar problems. Their story is just one example of the air pollution related health repercussions that are becoming increasingly prevalent.

These numbers are not merely statistics, but real people’s lives. For the Meyers, like for most American families, air pollution was not a daily concern. That changed when their 4 year old daughter, Amy, had her first asthma attack while playing at the playground close to where they lived in the Chicago area. As an energetic little girl, the swings and monkey bars had always been her favorite. After her diagnosis, it became difficult for her parents to keep her active and entertained, especially given their busy schedules as they both work in sales. A year later, their 11 year old son, Cody, developed asthma as well, preventing him from playing soccer, which he had always looked forward to after school. They soon realized that many of their neighbors were experiencing similar problems. Their story is just one example of the air pollution related health repercussions that are becoming increasingly prevalent.

Social Distance 1

The family in the passage seemed...

...not relatable at all ...very relatable

1 2 3 4 5 6 7

These numbers are not merely statistics, but real people’s lives. For the Washingtons, like for most American families, air pollution was not a daily concern. That changed when their 4 year old daughter, Tyra, had her first asthma attack while playing at the playground close to where they lived in the Chicago area. As an energetic little girl, the swings and monkey bars had always been her favorite. After her diagnosis, it became difficult for her parents to keep her active and entertained, especially given their busy schedules as they both work in sales. A year later, their 11 year old son, Terell, developed asthma as well, preventing him from playing soccer, which he had always looked forward to after school. They soon realized that many of their neighbors were experiencing similar problems. Their story is just one example of the air pollution related health repercussions that are becoming increasingly prevalent.

These numbers are not merely statistics, but real people’s lives. For the Meyers, like for most American families, air pollution was not a daily concern. That changed when their 4 year old daughter, Amy, had her first asthma attack while playing at the playground close to where they lived in the Chicago area. As an energetic little girl, the swings and monkey bars had always been her favorite. After her diagnosis, it became difficult for her parents to keep her active and entertained, especially given their busy schedules as they both work in sales. A year later, their 11 year old son, Cody, developed asthma as well, preventing him from playing soccer, which he had always looked forward to after school. They soon realized that many of their neighbors were experiencing similar problems. Their story is just one example of the air pollution related health repercussions that are becoming increasingly prevalent.

Social Distance 2

How willing would you be to have the family in the passage over for dinner?

...not willing at all ...very willing

1 2 3 4 5 6 7

These numbers are not merely statistics, but real people's lives. For the Washingtons, like for most American families, air pollution was not a daily concern. That changed when their 4 year old daughter, Tyra, had her first asthma attack while playing at the playground close to where they lived in the Chicago area. As an energetic little girl, the swings and monkey bars had always been her favorite. After her diagnosis, it became difficult for her parents to keep her active and entertained, especially given their busy schedules as they both work in sales. A year later, their 11 year old son, Terell, developed asthma as well, preventing him from playing soccer, which he had always looked forward to after school. They soon realized that many of their neighbors were experiencing similar problems. Their story is just one example of the air pollution related health repercussions that are becoming increasingly prevalent.

These numbers are not merely statistics, but real people's lives. For the Meyers, like for most American families, air pollution was not a daily concern. That changed when their 4 year old daughter, Amy, had her first asthma attack while playing at the playground close to where they lived in the Chicago area. As an energetic little girl, the swings and monkey bars had always been her favorite. After her diagnosis, it became difficult for her parents to keep her active and entertained, especially given their busy schedules as they both work in sales. A year later, their 11 year old son, Cody, developed asthma as well, preventing him from playing soccer, which he had always looked forward to after school. They soon realized that many of their neighbors were experiencing similar problems. Their story is just one example of the air pollution related health repercussions that are becoming increasingly prevalent.

Demographics

How old are you?

Are you...?

Male

Female

Other

What is the highest education level you have completed?

No high school degree

High school degree or equivalent (ie. GED)

Some college, no degree

Associate degree (ie. AA, AS)

Bachelors degree (ie. BA, BS)

Master's degree (ie. MA, MS)

Professional degree (ie. MD, DDS, DVM)

Doctorate (ie. PhD, EdD)

Powered by Qualtrics
